# Sexual selection and life history interact to influence the evolution of paternal care

**DOI:** 10.1002/ece3.70189

**Published:** 2024-08-21

**Authors:** Taya de Blonk, Isimeme N. Udu, Michael B. Bonsall, Hope Klug

**Affiliations:** ^1^ Department of Biology, Geology, and Environmental Science University of Tennessee at Chattanooga Chattanooga Tennessee USA; ^2^ Mathematical Ecology Research Group, Department of Biology University of Oxford Oxford UK; ^3^ St Peter's College, University of Oxford Oxford UK

**Keywords:** life history, mate preference, parental care, parental investment, paternal care, sex roles, sexual selection

## Abstract

Parental care is essential to offspring survival in many species. Understanding why males of some species provide care, whereas others do not, has received substantial attention. Previous research has found that sexual selection can favor paternal care, yet we still do not fully understand why sexual selection favors male care in some species but not others. It is also unclear when paternal care versus other preferred male trait(s) will be favored by sexual selection. We hypothesize that sexual selection can interact with basic life history to influence the conditions under which paternal care and/or another preferred male trait will be favored by sexual selection. We used a mathematical approach in which males alone provide parental care and exhibit a non‐care trait that is preferred in mate choice. Using this approach, we demonstrate that life‐history characteristics (stage‐specific mortality, fertilization success, gamete numbers) can interact with sexual selection to influence the evolution of paternal care and/or a preferred non‐care trait. In particular, whether (1) adult mortality, egg mortality, and fertilization success are high versus low and (2) a tradeoff exists between paternal care and a non‐care preferred trait will influence whether selection most strongly favors additional paternal care or a non‐care preferred trait. In general, we would expect strong selection for more male care when it is preferred in mate choice. In some cases, mate preferences for paternal care can inhibit selection for a preferred non‐care trait. Mate preferences for paternal care can also broaden the life‐history conditions under which we would expect the elaboration of male care to occur.

## Graphical abstract

Why do males of some species provide parental care, whereas paternal care is entirely absent in other species? Using a mathematical approach, we demonstrate that female mate preferences can interact with basic life history (mortality, fertilization success, etc.) to influence the evolution of paternal care. In some cases, mate preferences for paternal care can broaden the life‐history conditions under which we would expect higher levels of male care to evolve.

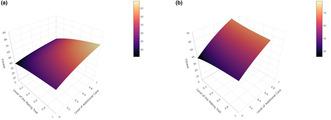

## 1 INTRODUCTION

Parental care has evolved independently multiple times within and across taxonomic groups and has a significant impact on offspring survival in many species (Balshine, 2012; Brown et al., 2010; Clutton‐Brock, 1991; Mank et al., 2005; Ostrovsky et al., 2009; Royle et al., 2012). Parental care is remarkably diverse, ranging from simple provisioning, grooming, and guarding behaviors to more distinctive forms of care such as matriphagy, filial cannibalism, and offspring abandonment (Clutton‐Brock, 1991; Davenport, 2018; Royle et al., 2012). Across taxa, there is a distinct variation in which sex provides care (Clutton‐Brock, 1991; Fromhage & Jennions, 2016; Henshaw et al., 2019; Kappeler et al., 2022; Kokko & Jennions, 2008; Liker et al., 2015; Royle et al., 2012). In general, parental care by males (i.e., paternal care) has been viewed as an evolutionary conundrum, as male fitness has historically been thought to be maximized by mating with as many females as possible (Andersson, 1994; Trivers, 1972). Despite this, male care is common in some animals, including some species of birds, mammals, fish, amphibians, and insects (reviewed in Clutton‐Brock, 1991; Royle et al., 2012).

While most research has focused on how natural selection favors parental care through increased offspring survival and quality (Alonso‐Alvarez & Velando, 2012; Klug & Bonsall, 2014), some empirical and theoretical research has highlighted the role that sexual selection plays in favoring paternal care (Alonzo, 2012; Araujo & Moura, 2023; Azad et al., 2022; Baylis, 1981; Forsgren, 1997; Fromhage et al., 2007; Künzler & Bakker, 2000; Lindström et al., 2006; Requena et al., 2013; Requena & Alonzo, 2017; Stiver & Alonzo, 2009; Tallamy, 1984, 2000). For example, paternal care is expected to evolve if males provide higher care quality than females and if females experience higher mortality during caring activities than males (Araujo & Moura, 2023). Similarly, parental effort is expected to increase when paternity confidence is high, although the link between paternity and paternal care is expected to weaken as care becomes less costly (Requena & Alonzo, 2017), and empirical studies suggest a complex relationship between paternity and paternal care (Alonzo, 2010). In addition, previous theoretical research suggests that paternal care can be favored evolutionarily when females preferentially mate with males that care (Alonzo, 2012). When paternal care is preferred in mate choice, males are not necessarily expected to experience a tradeoff between care and future mating opportunities, which can lead to increased selection for paternal care (Stiver & Alonzo, 2009).

Empirically, there is evidence that sexual selection influences paternal care. In the sand goby (*Pomatoschistus minutus*), males engage in more care in the presence of females (Pampoulie et al., 2004), and females preferentially mate with males that provide relatively high levels of care (Lindström et al., 2006), which in turn leads to higher rates of offspring survival (Forsgren, 1997). In one study, Lindström et al. (2006) experimentally manipulated the level of paternal care in the sand goby and performed dichotomous mate choice tests; consistently, females mated with males that exhibited higher levels of care, suggesting that sexual selection acts on paternal care in this system and that mate choice can select for levels of care beyond what natural selection alone would favor (Lindström et al., 2006). In the Neotropical harvestman (*Pseudopucrolia* sp.), guarding males were more attractive to females than non‐guarding males (Nazareth & Machado, 2010). In the glass frog (*Hyalinobatrachium cappellei*), males providing care were 70% more likely to receive additional eggs than males who were not providing care (Valencia‐Aguilar et al., 2020). Collectively, this theoretical and empirical research suggests that the origin, maintenance, and elaboration of paternal care can be influenced by sexual selection.

Despite a clear role for sexual selection in the evolution of paternal care, we still do not fully understand the conditions under which mate choice is most likely to favor paternal care. Why, for example, isn't male care more frequently the target of mate choice? Further, if male care—a trait that has the benefit of increasing offspring survival—is preferred by females, why would males ever exhibit a non‐care sexually selected trait? That is, if care has the dual fitness benefits of being preferred in mate choice and increasing offspring survival, why would a second (non‐care) trait also be favored? While previous theoretical work suggests that mate choice can favor the origin of care (Alonzo, 2012), it is also unclear when sexual selection, and mate preferences in particular, will favor additional male care once paternal care has arisen in a system. In general, additional work is needed to understand the role of sexual selection in driving patterns of paternal care, and it will be especially important to consider how sexual selection can influence the elaboration of (i.e., the increase in the level of) paternal care.

Basic life‐history traits (stage‐specific mortality, maturation, and reproductive rates) can strongly influence the likelihood that different forms of care will evolve (Klug et al., 2013a, 2013b), and in general, some form of parental care is expected to be more likely to evolve when offspring survive poorly in the absence of care, when parents have reduced future reproductive opportunities due to high adult mortality, and when eggs mature slowly (Klug & Bonsall, 2010). Similarly, mating dynamics can interact with life history to influence the origin of sex‐specific patterns of care (Azad et al., 2022). Given the important role of life history in driving evolutionary patterns of parental care, we hypothesize that basic life history (stage‐specific rates of mortality and reproductive parameters) can influence the conditions under which sexual selection is more or less likely to favor investment in male care versus other (non‐care) traits that are preferred in mate choice.

Here, we use a mathematical framework to explore how basic life history (stage‐specific mortality, reproductive parameters) and sexual selection due to female mate preferences interact to influence paternal care. We specifically examine how the fitness of male‐only care is affected by (1) basic life‐history parameters (egg and adult mortality, fertilization success, egg numbers) and (2) female preferences (no mate preferences, preferences for male care, and/or another male trait). In all scenarios, we assume that males provide some baseline level of care (i.e., paternal care has already originated), and males can then provide additional care beyond this baseline value (referred to herein as additional care). This additional care is either preferred or not preferred by females in mate choice. As such, our model focuses on the elaboration of male care once paternal care has arisen evolutionarily. In some cases, males also invest in and exhibit a non‐care trait that is preferred in mate choice (referred to herein as the mating trait). This trait can be thought of as any physical, chemical, or auditory trait that females prefer when selecting a mate. We consider four general sexual selection scenarios: there are no mate preferences (Scenario 1); additional male care is preferred in mate choice (Scenario 2); additional male care is not preferred in mate choice but the mating trait is exhibited and preferred in mate choice (Scenario 3); additional male care and a mating trait are both preferred in mate choice (Scenario 4) (Table 1). For each of these scenarios, we then ask how fitness varies with respect to basic life‐history parameter values (i.e., baseline egg and adult mortality, fertilization success, number of eggs). In doing so, we identify the basic life‐history conditions that are most likely to select for investment in additional paternal care (Scenarios 1–4) and/or investment in a preferred mating trait (Scenarios 3–4). We then identify how the optimal levels of investment in additional care and the mating trait vary in relation to each life‐history parameter for each scenario. Because no previous work explicitly considers how basic life history can interact with mate preferences to influence the elaboration of paternal care, our results provide a first look at how basic life history can interact with mating preferences to shape the fitness associated with paternal care and preferred mating traits.

**TABLE 1:** An overview of the tradeoffs across four scenarios in which there is/are: (1) no female mate preferences, (2) a female mate preference for additional male care, (3) a female mate preference for a male mating trait, and (4) female mate preferences for additional male care and a mating trait.

Sexual selection scenario	Males can provide additional paternal care	Males can exhibit mating trait	Tradeoffs
1: No mate preferences	Yes	No	Additional care is costly as it increases adult male mortality and is beneficial as it increases offspring survival
2: Females prefer additional care	Yes	No	Additional care is costly as it increases adult male mortality and is beneficial as it increases offspring survival. Additional care also increases male fertilization success due to the female preference
3: Females prefer mating trait	Yes	Yes	Additional care is costly as it increases adult male mortality and is beneficial as it increases offspring survival. The mating trait is costly as it increases male adult mortality and beneficial as it increases male fertilization success due to the female preference
4: Females prefer additional care and mating trait	Yes	Yes	Additional care is costly as it increases adult male mortality and is beneficial as it increases offspring survival. Additional care also increases male fertilization success due to the female preference. The mating trait is costly as it increases male adult mortality and beneficial as it increases male fertilization success due to the female preference

## 2 MATERIALS AND METHODS

### 2.1 Model overview

We use a mathematical model to explore how life‐history conditions influence the fitness associated with paternal care across different sexual selection scenarios. As mentioned above, males are assumed to provide some baseline level of parental care and can provide additional care that is either preferred (Scenarios 2 and 4) or not preferred (Scenarios 1 and 3) in mate choice (Table 1). Males in some cases exhibit a non‐care mating trait that is preferred in mate choice (Scenarios 3 and 4) (Table 1). We assume that males are the mate‐limited sex and hence experience sexual selection through mate preferences for additional care and/or the mating trait in Scenarios 2–4. Paternal care and the mating trait are associated with costs and/or benefits to the male (described below and in Table 1). The cost and benefit tradeoff functions of this model are described below, and graphical depictions are provided in the Figures S1–S14. In the modeling dynamics outlined below, we focus on male fitness and do not consider co‐evolutionary feedbacks among male and female dynamics, although the consideration of co‐evolution between male and female traits will be an important avenue of future research. For simplicity, we focus on paternal care of eggs, although the general dynamics below could equally apply to care beyond the egg stage. We additionally assume that males are in proximity to their young and capable of providing parental care.

### 2.2 Cost and benefit functions of male care and the male mating trait

In all scenarios, males provide some baseline level of care to their young. We use baseline egg mortality, *d*_*Eo*_ (i.e., the probability of egg death before accounting for additional care) as our proxy of initial male care; when baseline egg mortality is low, we assume that males provide a high level of baseline care, whereas when baseline egg mortality is high, males provide little baseline care to their young. Males can then provide additional care (*c*) to their eggs beyond this baseline level of care to increase further offspring survival. Both the baseline level of care and any additional care are costly to the adult males providing care, such that adult male mortality (i.e., the probability of adult male death, *d*_*A*_) increases as baseline egg mortality (*d*_*Eo*_) decreases and as additional care (*c*) increases:

(1) $$d_{A}=1-\left(1-d_{A0}\right)*\exp\left(-\left(\left(1-d_{E0}\right)+c\right)\right),$$

where *d*_*Ao*_ is the baseline male adult mortality (i.e., the probability that an adult male dies prior to accounting for costs of care). This cost of care occurs across all scenarios. In Scenarios 3 and 4, adult male mortality is also affected by investment in the mating trait, and the additive cost of the mating trait and additional care is described below.

Providing additional care is beneficial such that egg mortality (i.e., the probability of egg death after accounting for additional care, *d*_*E*_) decreases as additional care (*c*) increases, such that:

(2) $$d_{E}=d_{E0}∙\exp\left(-c\right),$$

where the parameters are as described above.

In Scenario 2, providing additional care is preferred in mate choice and, therefore, increases male fertilization success such that:

(3) $$r=1-\left(1-r_{0}\right)*\exp\left(-c\right),$$

where *r*_0_ is the baseline probability of egg fertilization (i.e., fertilization success prior to accounting for mate preferences) and *r* is the fertilization success after accounting for mate preferences.

In Scenario 3, the mating trait (but not care) is preferred in mate choice, and fertilization success, therefore, increases as the mating trait increases, such that:

(4) $$r=1-\left(1-r_{0}\right)*\exp\;\left(-m\right),$$

where the parameters are as described above.

In Scenario 4, both additional care and the mating trait are preferred in mate choice, and fertilization success increases as additional care and the mating trait increase, such that:

(5) $$r=1-\left(1-r_{0}\right)*\exp\left(-c+m\right),$$

where the parameters are as described above.

In Scenarios 3 and 4, investment in additional care and the mating trait is costly and increases adult mortality such that:

(6) $$d_{A}=1-\left(1-d_{A0}\right)*\exp\left(-\left(\left(1-d_{E0}\right)+m+c\right)\right),$$

where the parameters are as described above.

### 2.3 Male reproductive success and lifetime fitness

Reproductive success during a given reproductive episode, *R*, is determined by the number of limited opposite‐sex gametes present (i.e., the number of eggs available to fertilize, *b*), the probability those gametes are fertilized during mating (i.e., fertilization success, *r*), and the survival of those fertilized eggs, such that:

(7) $$R=r^{*}b^{*}\left(1-d_{E}\right).$$

Following Kokko (2007), the lifetime fitness of males providing additional care (Scenarios 1–4) and exhibiting the mating trait (Scenarios 2–4) is determined by current reproductive success during a given reproductive episode (Equation 7) and the expected reproductive success during future reproductive episodes, as well as the likelihood of surviving to those future reproductive episodes. We focus on adult male fitness and assume that males survive to their first reproductive episode. The likelihood of a male surviving through the first reproductive episode is (1 − *d*_*A*_), and the expected reproductive lifespan can be expressed as $\sum_{i=0}^{n}d_{A}{\left(1-d_{A}\right)}^{i}i$, where *i* = a given reproductive episode and *n* is the number of reproductive episodes (see Chapter 4 of Kokko (2007)). If male reproductive success during a given reproductive episode is *R* (Equation 7), then male lifetime fitness, *W*, is:

(8) $$W=R+R∙\sum_{i=0}^{n}d_{A}{\left(1-d_{A}\right)}^{i}i.$$

For each of the four scenarios outlined above, we calculated male fitness associated with investing in additional care (*c*) and/or a mating trait (*m*) in relation to basic life‐history parameters (i.e., baseline egg mortality, adult male mortality, fertilization success, the number of eggs present). We considered both the case in which *c + m* was constrained to a fixed value (0.5 in our analyses) and the case in which total investment in *c* and *m* varied across scenarios, such that *c =* 0.5 and *m =* 0.5. This means that for the constrained cases, *c =* 0.5 in Scenarios 1 and 2, and *c* and *m* each equal 0.25 in Scenarios 3 and 4; in the unconstrained cases *c =* 0.5 in Scenarios 1 and 2, and *c* and *m* each equal 0.5 in Scenarios 3 and 4. The unconstrained case is consistent with a lack of tradeoff between investment in additional care and the mating trait; that is, in the unconstrained case, investment in additional care does not affect investment in the mating trait and vice versa. The constrained case is consistent with a tradeoff between investment in additional care and the mating trait; that is, in the constrained case, investment in additional care does affect investment in the mating trait and vice versa, such that *c + m* is capped at 0.5. Importantly, investment in *c* and *m* is always associated with costs (discussed above), regardless of whether *c + m* is constrained to a fixed value or not.

These analyses allowed us to explore whether certain life‐history conditions are more or less likely to favor male investment in additional care and/or a mating trait across the four scenarios. For each scenario, we then used 3D plots to explore the relationship between male fitness and the level of additional care and/or the level of the mating trait in relation to each life‐history parameter, and these figures are presented in the Appendix A. This allowed us to determine if there were interactions between fitness and additional care or the mating trait and a given life‐history parameter. We also allowed *c* and *m* to vary continuously between zero and one to explore the sensitivity of the values chosen. All analyses were completed in R (R Core Team, 2023). Scripts are available at https://osf.io/2xfb9/.

We hypothesized that mate preferences can favor the evolution of additional care and a mating trait but this effect of sexual selection on care will depend on the life‐history values (e.g., egg and adult mortalities, fertilization success, number of eggs) considered. We hypothesized when care is not preferred in mate choice, the life‐history conditions that favor care could differ from the life‐history conditions that favor the preferred mating trait. Lastly, we hypothesized that when care is preferred in mate choice, the life‐history conditions that favor additional care could differ from the life‐history conditions that favor the mating trait.

## 3 RESULTS

### 3.1 Baseline egg mortality

#### 3.1.1 Total trait investment varies across scenarios such that investment in additional care and the mating trait do not trade off

**FIGURE 1:**
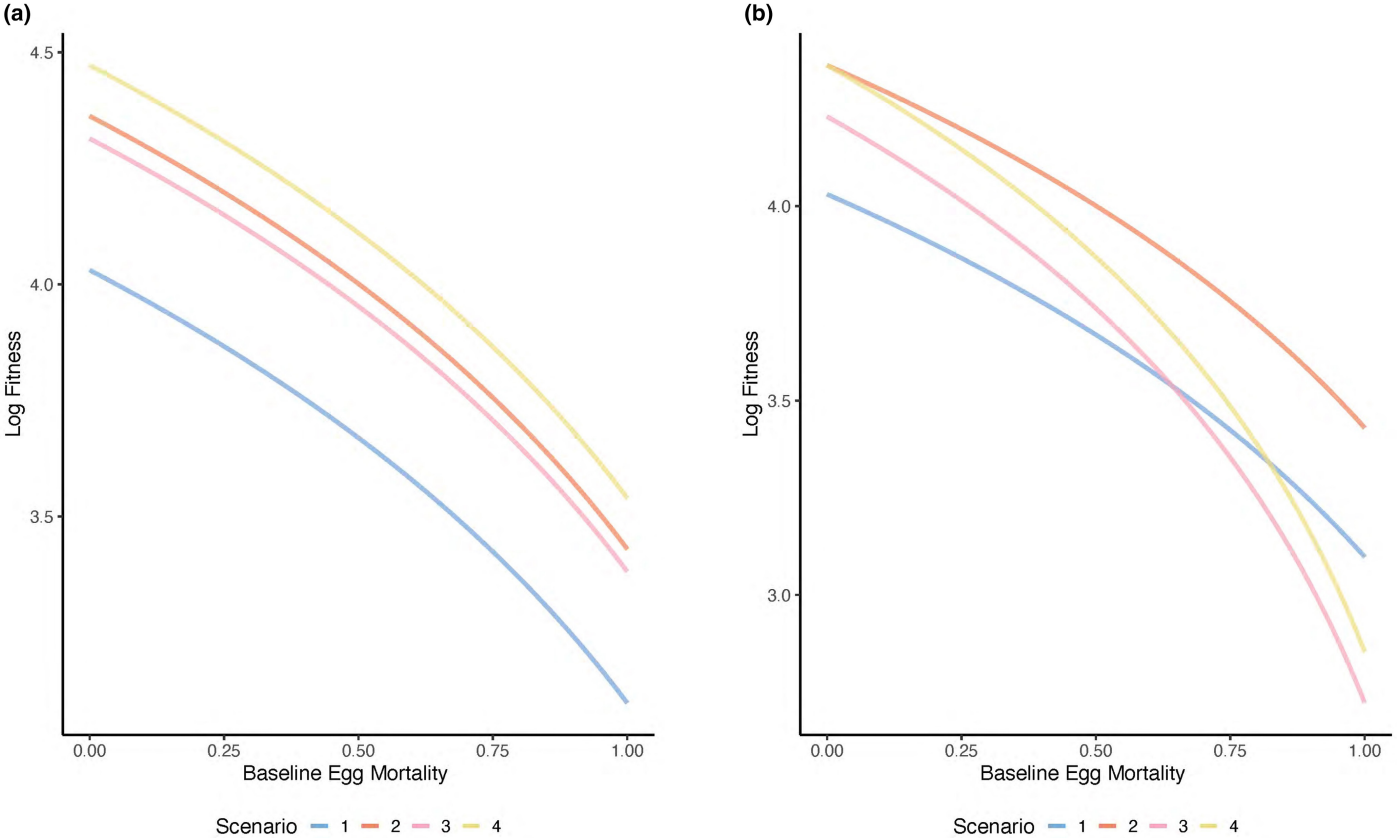
The effect of baseline egg mortality, which is a measure of baseline male care, on male lifetime fitness across mate preference and paternal care scenarios. In (a) total investment in additional care and a mating trait is unconstrained (*c* = 0.5 in Scenarios 1–4 and *m* = 0.5 in Scenarios 3–4), and in (b) total investment in additional care and a mating trait is constrained (*c + m* = 0.5 in all scenarios). Log (fitness) is shown for each of four scenarios: (1) additional paternal care occurs but mate preferences are absent (Scenario 1, blue line), (2) additional paternal care occurs and is preferred in mate choice (Scenario 2, orange line), (3) additional paternal care is present but not preferred and a male mating trait is present and preferred (Scenario 3, pink line), and (4) both additional paternal care and a mating trait are present and preferred in mate choice (Scenario 4, yellow line). Other parameter values are as follows and as described in the text: *b* = 100, *d*_*Ao*_ = 0.5, *r*_*o*_ = 0.5, *n* = 5.0.

As baseline egg mortality increases, fitness decreases for all scenarios when total investment in additional care and a mating trait is not constrained to a fixed value (i.e., when *c* = 0.5 in Scenarios 1–4 and *m* = 0.5 in Scenarios 3–4, such that total trait investment is either 0.5 or 1; Figure 1a). The pattern of lifetime fitness decreasing as egg mortality increases is intuitive as individuals have lower fitness when offspring have high mortality. Importantly, the slopes of the relationship between baseline egg mortality and fitness do not differ across the scenarios considered (Figure 1a). This suggests that the baseline egg mortality, which is the measure of the baseline level of care provided by males in our model, will not influence which scenario has the highest (or lowest) fitness when total investment in additional care and a mating trait is not constrained to a fixed value (i.e., when investment in additional care and the mating trait do not trade off; Figure 1a).

When investment in additional care and the mating trait is not constrained to a fixed value, fitness will be greatest for the scenario in which both additional care and a male mating trait are preferred across all baseline egg mortality values (Figure 1a, yellow line), suggesting that investing in both additional care and a preferred mating trait will be strongly selected for if females prefer both additional care and the mating trait. The scenario in which additional care is preferred and a male mating trait is absent has the second highest fitness (Figure 1a, orange line), whereas the scenario in which additional care is present but only the mating trait is preferred has the third highest fitness (Figure 1a, pink line) across baseline egg mortality values. Notably, the fitness difference between these two scenarios (Scenarios 2 and 3) is minimal, suggesting that selection for additional care or a preferred mating trait can be similar. The scenario in which additional care occurs and in which there are no mate preferences has the lowest fitness across egg mortality values (Figure 1a, blue line).

These results suggest that: (1) both additional care and a mating trait can be strongly selected for and result in relatively high fitness when mate preferences for care and a mating trait exist (Figure 1a, yellow line); (2) it is beneficial to invest in both additional care and a mating trait when mate preferences for both additional care and a mating trait exist; (3) if there is only preference for one trait, male fitness will be higher if additional care is preferred (Figure 1a, orange vs. pink line); and (4) the fitness of investing in additional care will be relatively low when mate preferences are absent in comparison to cases in which mate preferences are present (Figure 1a, blue line vs. pink, orange, and yellow lines). Importantly, these patterns are consistent across all baseline egg mortality values considered (Figure 1a), suggesting that baseline egg mortality will not determine which mate‐preference scenario has highest fitness when total trait investment is not constrained.

#### 3.1.2 Total trait investment is fixed across scenarios such that investment in additional care and the mating trait trade off

Consistent with the above results (Figure 1a), when total investment in additional care and a mating trait is constrained to a fixed value (i.e., when *c* + *m* = 0.5), fitness decreases as baseline egg mortality increases for all scenarios (Figure 1b). In contrast to the above results (Figure 1a), when trait investment is constrained to a fixed value, baseline egg mortality will influence the relative fitness of Scenarios 1–4 (Figure 1b).

At very low baseline egg mortality, fitness is highest when (1) additional care alone (Figure 1b, orange line) and (2) additional care and a mating trait are preferred in mate choice (Figure 1b, yellow line). As baseline egg mortality increases, it quickly becomes most beneficial to invest solely in additional care if it is preferred in mate choice (Figure 1b, orange line), and the fitness associated with investing in both additional care and a mating trait quickly decreases (Figure 1b, yellow line). At low‐to‐moderate values of baseline egg mortality, the scenario in which additional care occurs but only the mating trait is preferred has the third highest fitness (Figure 1b, pink line), followed by the scenario in which additional care occurs but mate preferences are absent (Figure 1b, blue line).

At relatively high levels of baseline egg mortality, investing in additional care when it is preferred continues to have the highest fitness (Figure 1b, orange line). However, at high values of baseline egg mortality, the strategy with the second highest fitness is investment in additional care when no mate preferences exist (Figure 1b, blue line), followed by investment in additional care and a mating trait when they are both preferred (Figure 1b, yellow line). At high values of baseline egg mortality, investing in a mating trait and additional care will be associated with the lowest fitness when there is only a preference for the mating trait (Figure 1b, pink line).

These results suggest that when total investment in additional care and a mating trait are constrained to a fixed value (i.e., when investment in additional care and a preferred mating trait trade off) and eggs are not expected to survive well without additional care, investment in a mating trait over additional care will be selected against. That is, when total trait investment is constrained to a fixed value, high egg mortality (and low levels of baseline male care) can inhibit investment into a non‐care sexually selected trait (Figure 1b, yellow and pink lines).

In summary, baseline egg mortality will influence the scenario associated with the highest fitness when investment into additional care and a mating trait is constrained to a fixed value, that is, when investment in additional care and the mating trait trade off. When total investment is constrained to a fixed value and egg mortality is high, investing in a mating trait is likely to be selected against in favor of additional investment in care. In contrast, when investment into additional care and a mating trait is not constrained to a fixed value, baseline egg mortality will not influence the strategy that is associated with the greatest fitness.

### 3.2 Fertilization success

#### 3.2.1 Total trait investment varies across scenarios such that investment in additional care and the mating trait do not trade off

**FIGURE 2:**
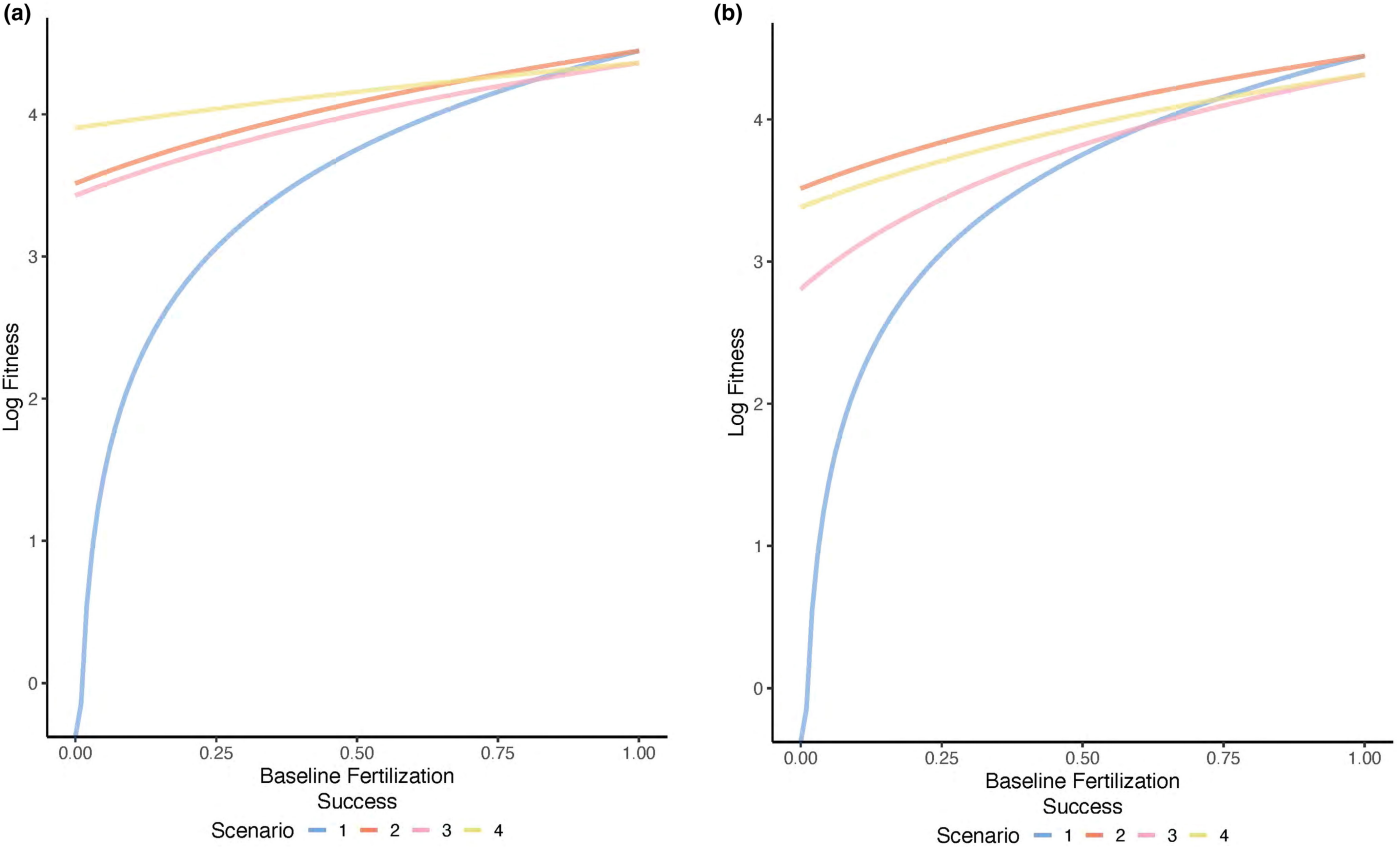
The effect of baseline egg fertilization success on male lifetime fitness across mate preference and paternal care scenarios. In (a) total investment in additional care and a mating trait is unconstrained (*c* = 0.5 in Scenarios 1–4 and *m* = 0.5 in Scenarios 3–4), and in (b) total investment in additional care and a mating trait is constrained (*c + m* = 0.5 in all scenarios). Fitness is shown for each of four scenarios: (1) additional paternal care occurs but mate preferences are absent (Scenario 1, blue line), (2) additional paternal care occurs and is preferred in mate choice (Scenario 2, orange line), (3) additional paternal care is present but not preferred and a male mating trait is present and preferred (Scenario 3, pink line), and (4) both additional paternal care and a mating trait are present and preferred in mate choice (Scenario 4, yellow line). Other parameter values are as follows and as described in the text: *b* = 100, *d*_*Ao*_ = 0.5, *d*_*Eo*_ = 0.5, *n* = 5.0.

When total investment in additional care and the mating trait is not constrained to a fixed value, fitness increases as baseline fertilization success increases for all scenarios (Figure 2a). That is, higher fertilization success will result in a higher fitness, which is intuitive given that greater egg fertilization will lead to a greater number of offspring produced, all else equal. Importantly, the scenario with the greatest fitness depends on baseline fertilization success. When additional care and/or the mating trait are preferred (Scenarios 2–4), baseline fertilization success has relatively little impact on fitness (Figure 2a, yellow, orange, pink lines). This occurs as preferences for additional care or the mating trait increase the realized fertilization success, and hence overall fitness, across the range of baseline fertilization values. When preferences for either trait are absent (Scenario 1), fitness is initially negative but rapidly increases as baseline fertilization success increases (Figure 2a, blue line). At low‐to‐moderate levels of baseline fertilization success, the scenario in which mate preferences are absent has the lowest fitness (Scenario 1) (Figure 2a, blue line), and the scenario in which there is a preference for additional care and a mating trait has the greatest fitness (Figure 2a, yellow line). In contrast, at high levels of baseline fertilization success, fitness is greatest when there is only a preference for additional care (Figure 2a, orange line) or when there is no preference for either a mating trait or additional care (Figure 2a, blue line). This suggests that investing solely in additional care, even when it is not preferred in mate choice, is more beneficial for fitness than investing into a mating trait if baseline fertilization success is high. This pattern occurs as the additional benefits of investing in a preferred mating trait will be relatively small when baseline fertilization success is high. Thus, when baseline fertilization success is high, males benefit minimally from mate preferences for either trait. Instead, investment in a trait that increases offspring fitness (i.e., additional care) will be associated with the greatest fitness.

In summary, baseline fertilization success influences the strategy with the highest fitness when total investment is not constrained to a fixed value, that is, when investment in additional care and the mating trait do not trade off. While investment in additional care and a mating trait are likely to be selected for at low levels of baseline fertilization success, investing in a mating trait is less likely to be selected for at high levels of baseline fertilization success.

#### 3.2.2 Total trait investment is fixed across scenarios such that investment in additional care and the mating trait trade off

Fitness increases as baseline fertilization success increases for all scenarios when total investment in additional care and a mating trait is constrained to a fixed value (Figure 2b). Similar to the scenarios in which total investment is unconstrained (Figure 2a), when care and/or the mating trait are preferred, baseline fertilization success has relatively little impact on fitness (Figure 2b, orange, yellow, pink lines). Similarly, when there are no mate preferences for either trait, fitness is initially negative and rapidly increases as baseline fertilization success increases (Figure 2b, blue line). At low‐to‐moderate levels of baseline fertilization success, the scenario in which mate preferences are absent has the lowest fitness (Figure 2b, blue line), the scenario in which additional care is preferred has the greatest fitness (Figure 2b, orange line), and the scenario in which additional care and the mating trait are preferred has the second highest fitness (Figure 2b, yellow line). The scenario in which only the mating trait is preferred has the third‐highest fitness (Figure 2b, pink line). These results suggest that when total investment is constrained to a fixed value, that is, when investment in additional care and the mating trait trade off, and baseline fertilization success is low‐to‐moderate, investment in additional care that is preferred in mate choice will have the greatest fitness.

At relatively high levels of baseline fertilization success, investing in additional care, regardless of whether care is preferred or not (Figure 2b, blue and orange lines), will have the greatest fitness. This pattern occurs because the benefits of investing in a preferred mating trait will be relatively small when baseline fertilization success is already high.

Collectively, these results suggest that baseline fertilization success will influence the scenario that is associated with the greatest fitness. When trait investment is not constrained to a fixed value and preferences for additional care and the mating trait exist, investing in both additional care and a mating trait can be strongly selected for at low‐to‐moderate levels of fertilization success. In contrast, investment in only additional care, regardless of whether there is a preference for care, will be associated with the greatest fitness when baseline fertilization success is high. When total trait investment is constrained to a fixed value, investing in only additional care when mate preferences for care exist will be most strongly selected for across levels of baseline egg fertilization success. Thus, when baseline egg fertilization success is high, selection is likely to act against investment in a preferred mating trait in favor of investment in additional care.

### 3.3 Number of female gametes

#### 3.3.1 Total trait investment varies across scenarios such that investment in additional care and the mating trait do not trade off

**FIGURE 3:**
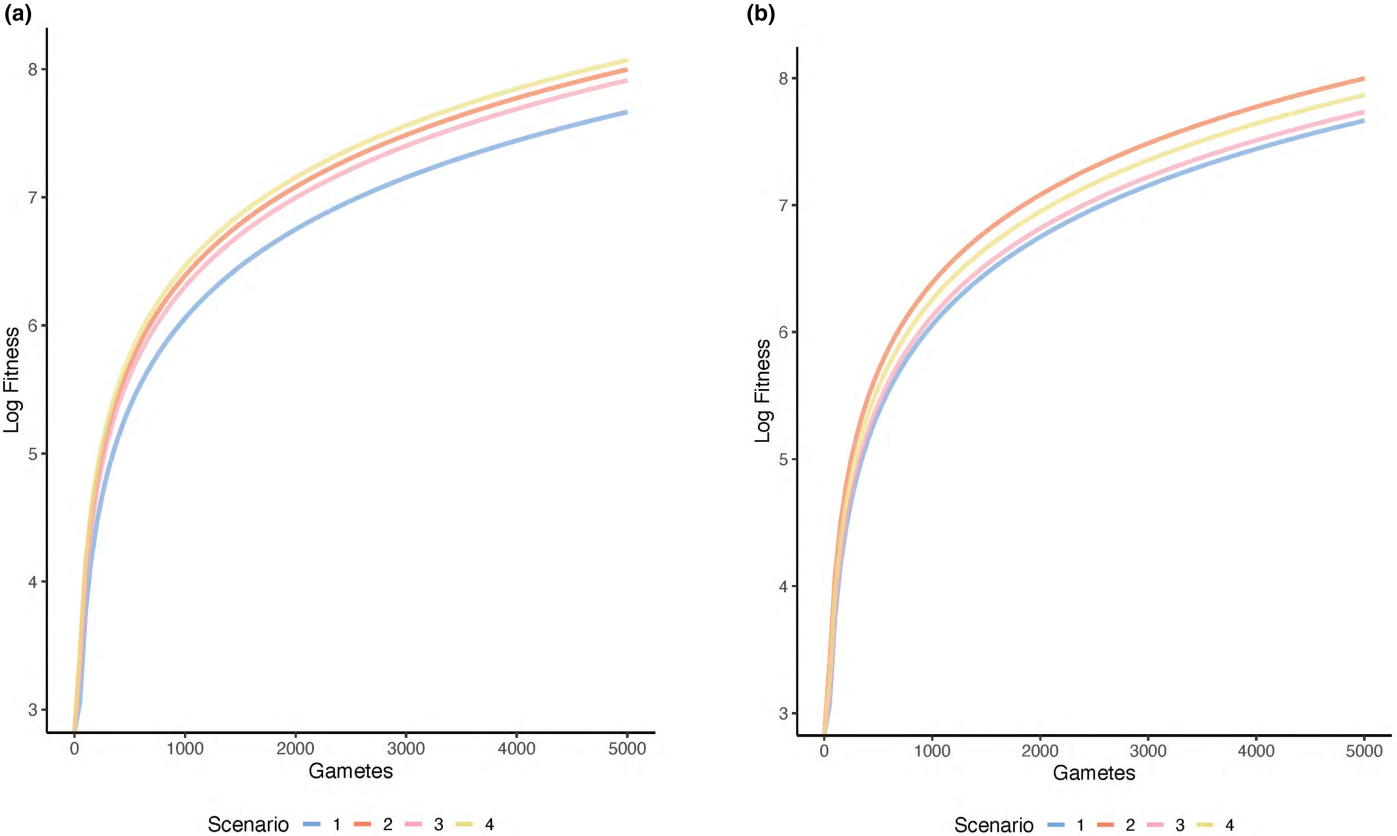
The effect of the number of reproductively available female gametes on male lifetime fitness across mate preference and paternal care scenarios. In (a) total investment in additional care and a mating trait is unconstrained (*c* = 0.5 in Scenarios 1–4 and *m* = 0.5 in Scenarios 3–4), and in (b) total investment in additional care and a mating trait is constrained (*c + m* = 0.5 in all scenarios). Fitness is shown for each of four scenarios: (1) additional paternal care occurs but mate preferences are absent (Scenario 1, blue line), (2) additional paternal care occurs and is preferred in mate choice (Scenario 2, orange line), (3) additional paternal care is present but not preferred and a male mating trait is present and preferred (Scenario 3, pink line), and (4) both additional paternal care and a mating trait are present and preferred in mate choice (Scenario 4, yellow line). Other parameter values are as follows and as described in the text: *r*_*o*_ = 0.5, *d*_*Ao*_ = 0.5, *d*_*Eo*_ = 0.5, *n* = 5.0.

As the number of female gametes increases in the population, fitness increases across all scenarios (Figure 3a), and this will lead to greater reproductive success. The slope of the relationship between the number of female gametes and fitness is invariant across scenarios considered when investment in additional care and a mating trait is not constrained to a fixed value (Figure 3a), suggesting that the number of female gametes will not influence which scenario has the highest (or lowest) fitness.

When total investment is not constrained, the scenario in which both additional care and a mating trait are preferred has the highest fitness regardless of number of female gametes (Figure 3a, yellow line). This suggests that overall greater investment into preferred traits is beneficial to males if such investment is possible. The scenario in which additional care is preferred and a male mating trait is absent has the second highest fitness (Figure 3a, orange line), while the scenario in which only a mating trait is preferred has the third highest fitness (Figure 3a, pink line). The scenario in which additional care occurs when there are no mate preferences has the lowest fitness (Figure 3a, blue line). These results suggest that regardless of the number of female gametes, investment into additional care and a mating trait can be strongly selected for if mate preferences for both traits exist (Figure 3a, yellow line). Likewise, fitness will be higher if additional care is preferred when only one of the traits is preferred (Figure 3a, orange vs. pink line). These patterns are consistent across the range of female gamete numbers considered (Figure 3a), suggesting that the number of female gametes alone does not directly favor one scenario over another.

#### 3.3.2 Total trait investment is fixed across scenarios such that investment in additional care and the mating trait trade off

As the number of female gametes increases, fitness increases across all scenarios when total investment is constrained to a fixed value (Figure 3b). Similarly, the slope of the relationship between the number of female gametes and fitness does not differ across scenarios when total investment is constrained (Figure 3b). These patterns are consistent with the case in which total investment is not constrained (Figure 3a).

When total investment in additional care and the mating trait is constrained to a fixed value, the scenario in which only additional care is preferred has the highest fitness across female gamete numbers (Figure 3a, orange line), suggesting that investment in additional care will be strongly selected when it is preferred in mate choice. The scenario in which both additional care and a mating trait are preferred has the second highest fitness (Figure 3b, yellow line), while the scenario in which only a mating trait is preferred has the third highest fitness (Figure 3b, pink line). The scenario in which additional care occurs with no mate preferences has the lowest fitness (Figure 3b, blue line). These results suggest that (1) investment in additional care will be strongly selected when care is the only preferred trait (Figure 3b, orange line), and (2) fitness will be higher if additional care and the mating trait are preferred versus if only the mating trait or neither are preferred (Figure 3b, yellow line versus pink and blue lines). These patterns are consistent across the range of female gamete numbers (Figure 3b), such that the number of female gametes does not influence which scenario will have the highest fitness.

In summary, regardless of whether total trait investment is constrained versus not, the number of female gametes will not influence the relative fitness of the scenarios.

### 3.4 Baseline adult male mortality

#### 3.4.1 Total trait investment varies across scenarios such that investment in additional care and the mating trait do not trade off

**FIGURE 4:**
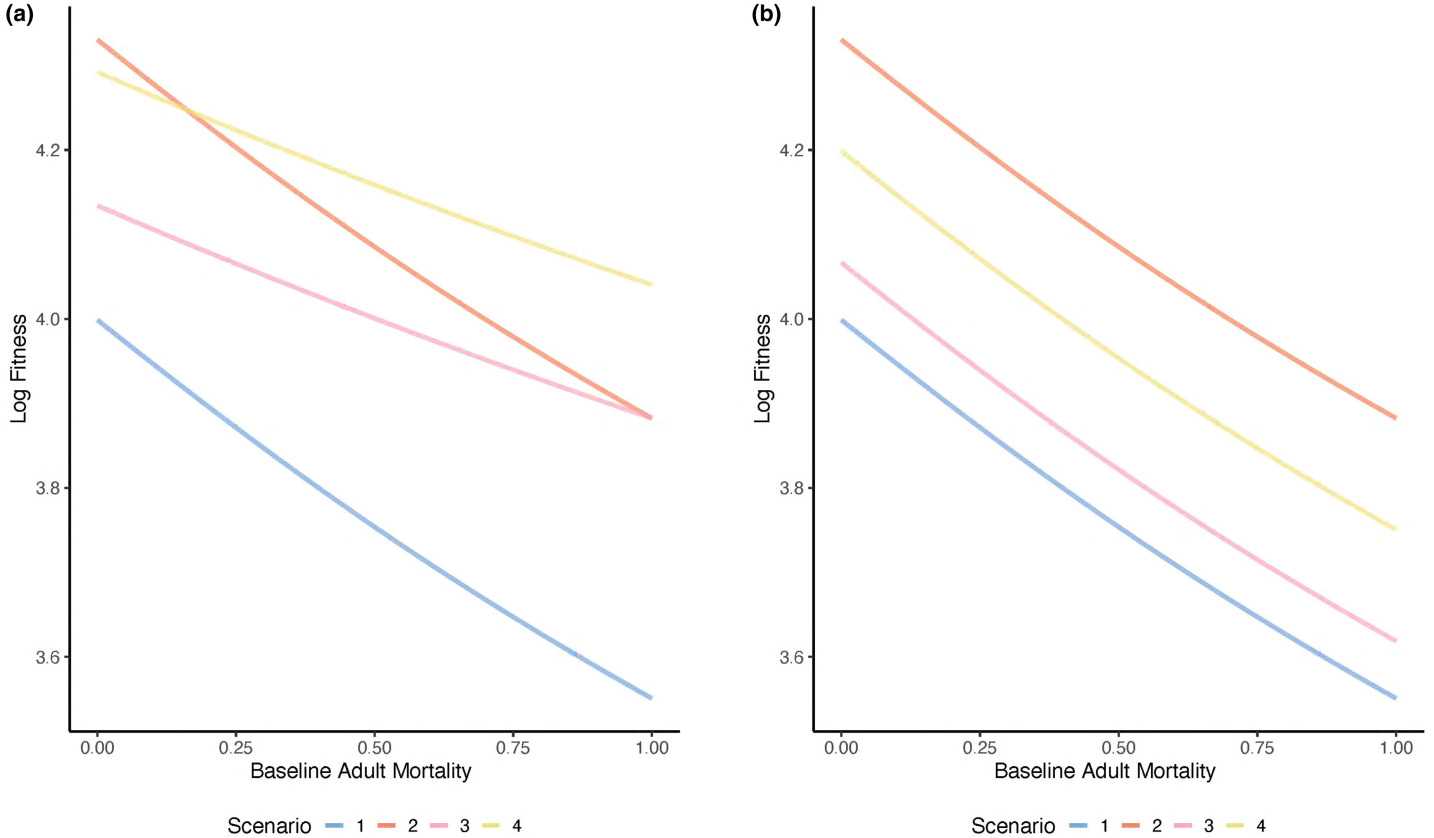
The effect of baseline adult mortality on male lifetime fitness across mate preference and paternal care scenarios. In (a) total investment in additional care and a mating trait is unconstrained (*c* = 0.5 in Scenarios 1–4 and *m* = 0.5 in Scenarios 3–4), and in (b) total investment in additional care and a mating trait is constrained (*c + m* = 0.5 in all scenarios). Fitness is shown for each of four scenarios: (1) additional paternal care occurs but mate preferences are absent (Scenario 1, blue line), (2) additional paternal care occurs and is preferred in mate choice (Scenario 2, orange line), (3) additional paternal care is present but not preferred and a male mating trait is present and preferred (Scenario 3, pink line), and (4) both additional paternal care and a mating trait are present and preferred in mate choice (Scenario 4, yellow line). Other parameter values are as follows and as described in the text: *r*_*o*_ = 0.5, *b* = 100, *d*_*Eo*_ = 0.5, *n* = 5.0.

Fitness decreases as baseline adult male mortality increases for all scenarios (Figure 4a). This is intuitive as individuals have lower fitness if they are more likely to die. The relative fitness of the scenarios depends on baseline adult mortality. At very low levels of baseline adult mortality, investing in additional care when care is preferred (Figure 4a, orange line) has the greatest fitness, and investing in both additional care and a mating trait when both are preferred has the second highest fitness (Figure 4a, yellow line). At moderate and high values of baseline adult mortality, it becomes more beneficial to invest in both additional care and the mating trait when preferences for both exist (Figure 4a, yellow line), and investing in only additional care when there is a preference for care alone is associated with the second highest fitness (Figure 4a, orange line). In general, this suggests that at higher levels of baseline adult male mortality, fitness is greatest if males invest relatively more into preferred traits, that is, both the mating trait and additional care.

Investing in additional care and the mating trait when only the mating trait is preferred has the third highest fitness across levels of baseline adult mortality (Figure 4a, pink line), and investing in additional care when no preferences exist has the lowest fitness (Figure 4a, blue line).

In summary, baseline adult male mortality influences the relative fitness of strategies when total investment is unconstrained. At very low levels of baseline adult male mortality, investing in only additional care when care is preferred will be most strongly selected for, whereas at moderate and high levels of baseline adult mortality, investing in both additional care and a mating trait when both are preferred will have the greatest fitness.

#### 3.4.2 Total trait investment is fixed across scenarios such that investment in additional care and the mating trait trade off

When investment in additional care and the mating trait is constrained, fitness decreases as baseline adult mortality increases for all scenarios (Figure 4b). When total trait investment is constrained, the slopes of the relationship between baseline adult mortality and fitness do not differ across scenarios (Figure 4b). Across levels of baseline adult mortality, investing in additional care when care is preferred has the greatest fitness (Figure 4b, orange line), investing in both additional care and a mating trait when both are preferred has the second highest fitness (Figure 4b, yellow line), and investing in additional care and the mating trait when only the mating trait is preferred has the third highest fitness (Figure 4b, pink line). Investing in additional care when no preferences exist has the lowest fitness (Figure 4b, blue line).

In summary, when total trait investment is constrained, baseline adult mortality will not influence which scenario has the highest (or lowest) fitness. Across levels of baseline adult mortality, fitness will be greatest when males invest only in additional care if care is preferred (Figure 4b, orange line).

### 3.5 Level of additional care and the mating trait

**FIGURE 5:**
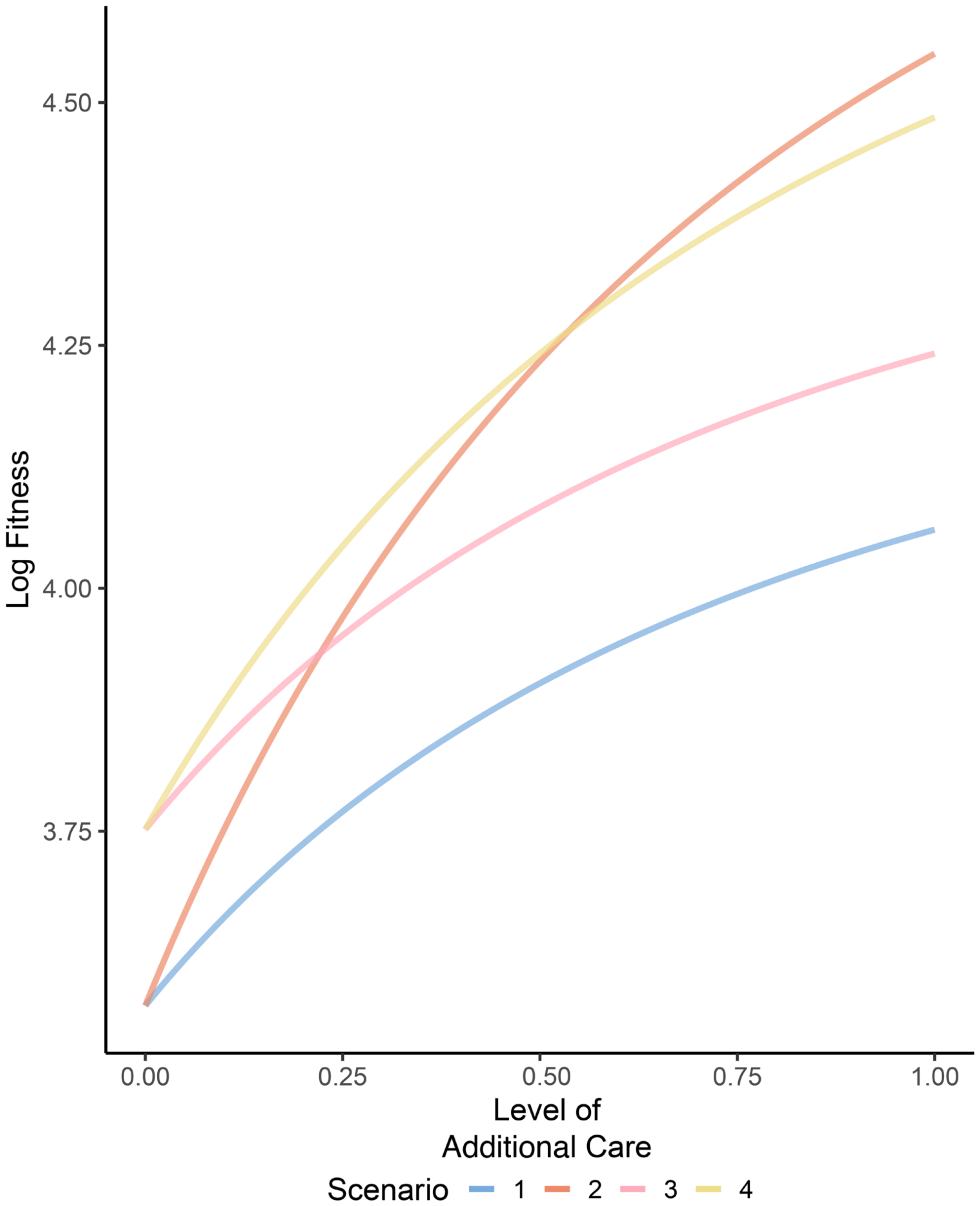
The effect of additional care level on male lifetime fitness across mate preference and paternal care scenarios. Fitness is shown for each of four scenarios: (1) additional paternal care occurs but mate preferences are absent (Scenario 1, blue line), (2) additional paternal care occurs and is preferred in mate choice (Scenario 2, orange line), (3) additional paternal care is present but not preferred and a male mating trait is present and preferred (Scenario 3, pink line), and (4) both additional paternal care and a mating trait are present and preferred in mate choice (Scenario 4, yellow line). Other parameter values are as follows and as described in the text: *m* = 0.5, *r*_*o*_ = 0.5, *b* = 100, *d*_*Eo*_ = 0.5, *n* = 5.0, *d*_*Ao*_ = 0.5.

We next explored the effect of varying levels of additional care on male fitness while holding the level of the male mating trait to a fixed value. Fitness for all scenarios increased as additional care increased when baseline egg mortality is moderately high (*d*_*Eo*_ = 0.5) (Figure 5). This pattern occurs because regardless of the scenario considered, additional care increases offspring survival. When investment in additional care is relatively low (<0.5) and investment in the mating trait is equal to 0.5, fitness will be greatest when males invest in both additional care and the mating trait if both are preferred (Figure 5, yellow line). In contrast, when investment in additional care is relatively high (>0.5) and there is only a preference for care, fitness will be greatest when males invest only in additional care (Figure 5, orange line). Fitness will be lowest when males invest in additional care if a preference for additional care is absent across scenarios (Figure 5, blue line).

**FIGURE 6:**
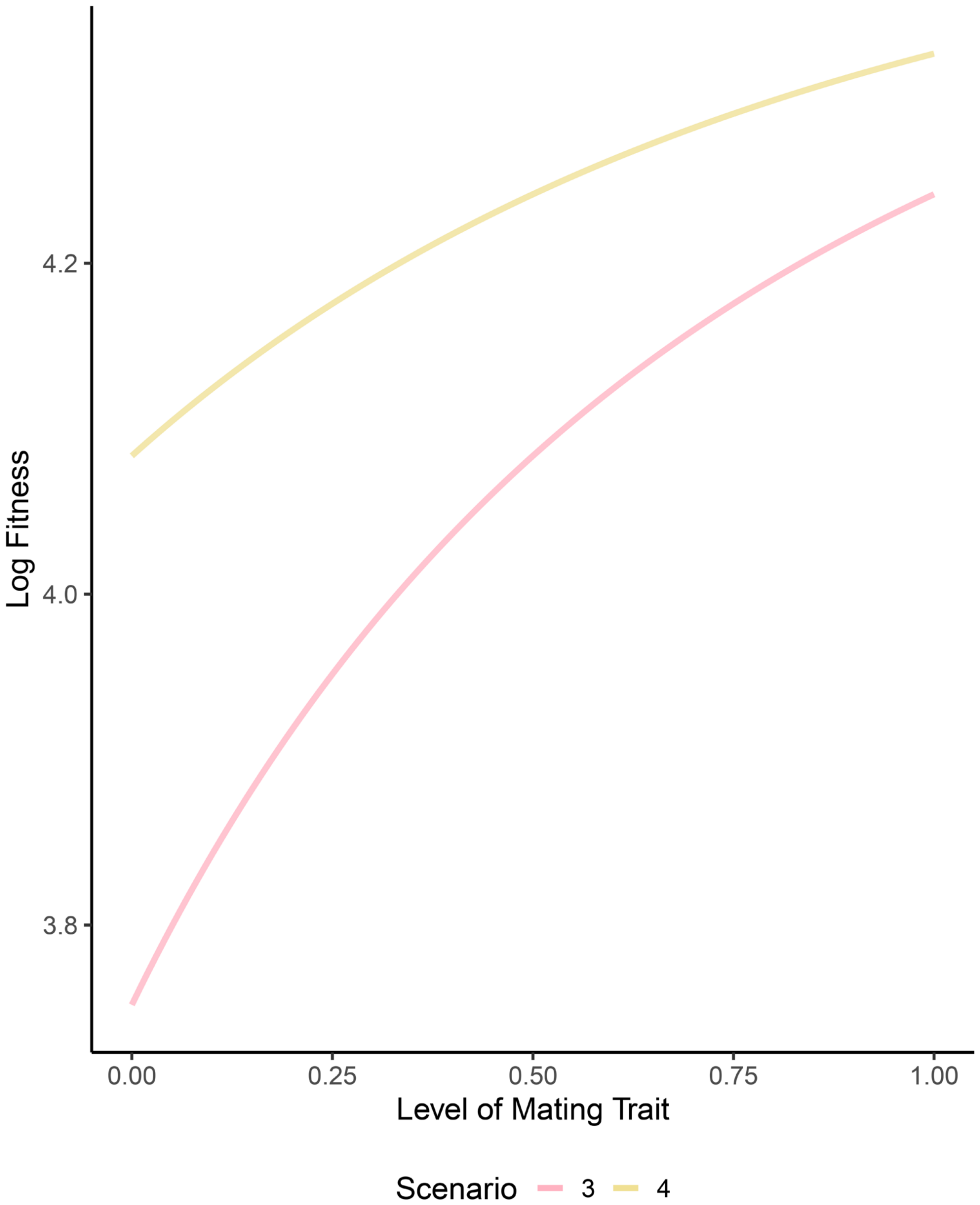
The effect of mating trait level on male lifetime fitness across mate preference scenarios. Fitness is shown for each of two scenarios: (1) additional paternal care is present but not preferred and a male mating trait is present and preferred (Scenario 3, pink line), and (2) both additional paternal care and a mating trait are present and preferred in mate choice (Scenario 4, yellow line). Other parameter values are as follows and as described in the text: *c* = 0.5, *r*_*o*_ = 0.5, *b* = 100, *d*_*Eo*_ = 0.5, *n* = 5.0, *d*_*Ao*_ = 0.5.

We then explored the effect of varying levels of the mating trait on male fitness while holding the level of additional care to a fixed value. When parents invest in a mating trait (Scenarios 3–4) and baseline egg mortality is moderately high (*d*_*Eo*_ = 0.5), fitness increases as investment in the mating trait increases (Figure 6). Investing in both the mating trait and additional care has greater fitness when both are preferred (Figure 6, yellow line) relative to the scenario in which only the mating trait is preferred (Figure 6, pink line).

**FIGURE 7:**
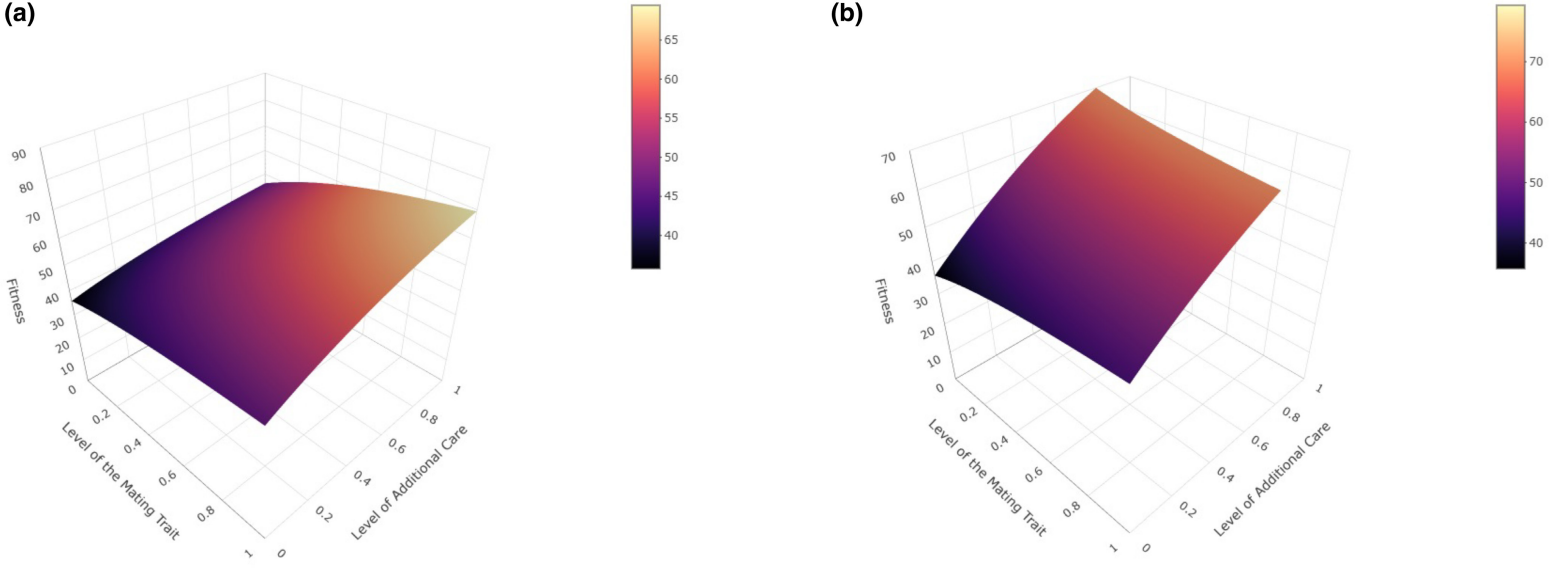
The effect of additional care and the mating trait levels on male lifetime fitness across mate preference scenarios. Fitness is shown for each of two scenarios: (a) additional paternal care is present but not preferred and a male mating trait is present and preferred (Scenario 3), and (b) both additional paternal care and a mating trait are present and preferred in mate choice (Scenario 4). Other parameter values are as follows and as described in the text: *r*_*o*_ = 0.5, *b* = 100, *d*_*Ao*_ = 0.5, *n* = 5.0, *d*_*Ao*_ = 0.5.

We also examined the relationship between the mating trait, additional care, and fitness when both additional care and the mating trait were allowed to vary. When only the mating trait is preferred, fitness increases as the mating trait and additional care increases (Figure 7a). If both additional care and the mating trait are preferred, fitness is highest when the level of additional care is high and the level of the mating trait is low (Figure 7b). In general, fitness increases as the levels of additional care and the mating trait increase, which is consistent with the results presented in Figures 5 and 6. However, investment in additional care is more beneficial (i.e., it has a great impact on fitness) than investment in the mating trait (Figure 7b).

### 3.6 Interactions between trait investment, life history, and fitness across scenarios

**FIGURE A1:**
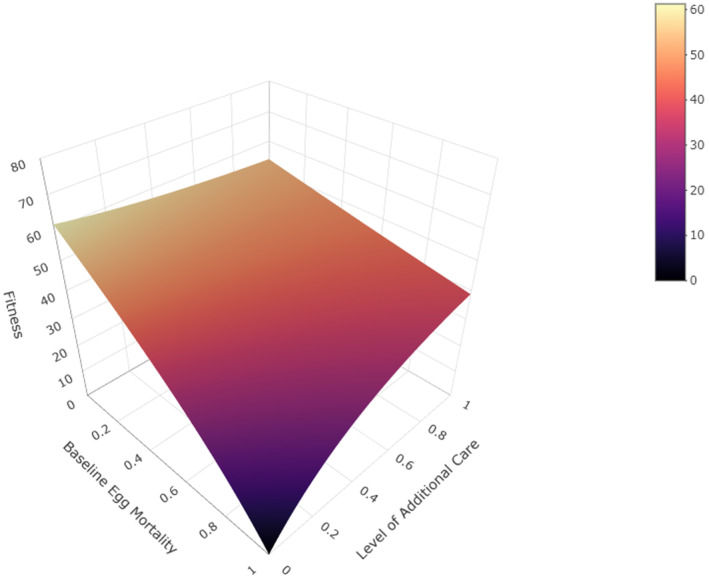
Scenario 1: The relationship between baseline egg mortality, level of care, and fitness. For Scenario 1 (no mate preferences), fitness decreases as baseline egg mortality increases. When baseline egg mortality is very low, fitness increases as the level of care decreases; this pattern occurs because there are minimal benefits of care when offspring survive well in the absence of care. When baseline egg mortality is high, fitness increases as the level of care increases, as these are the conditions under which offspring need care the most. The findings that fitness increases as baseline egg mortality decreases and that fitness increases as the level of care increases when baseline egg mortality is high are consistent with the results presented in Figures 1 and 5. However, the results in the current figure highlight that lower levels of care will be selected for if offspring survive well without care if there are no preferences for care.

**FIGURE A2:**
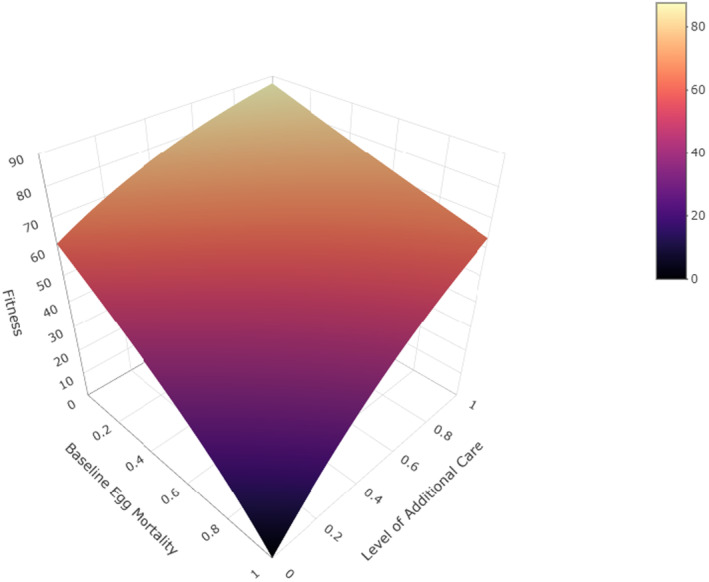
Scenario 2: The relationship between baseline egg mortality, level of care, and fitness. For Scenario 2 (mate preference for care), fitness increases as baseline egg mortality decreases and as the level of care increases. These findings are consistent with the results presented in Figures 1 and 5 and suggest that mate preferences for care can strongly select for parental care.

**FIGURE A3:**
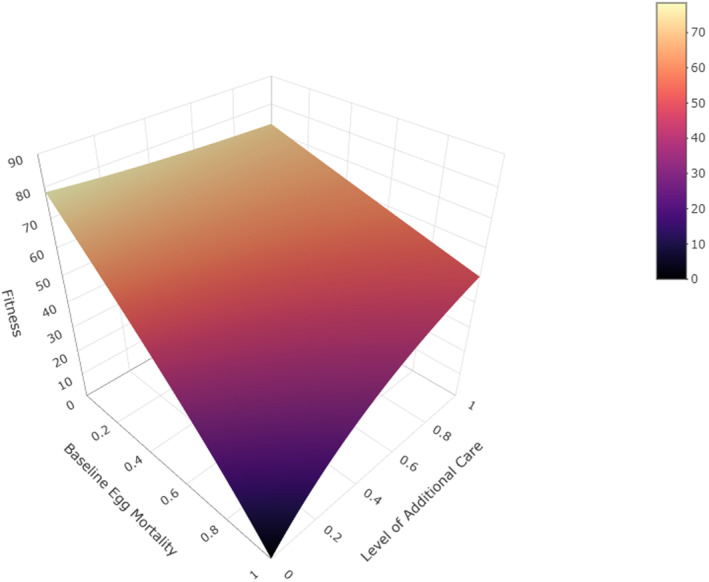
Scenario 3: The relationship between baseline egg mortality, level of care, and fitness. For Scenario 3 (mate preference for mating trait), fitness decreases as baseline egg mortality increases. When baseline egg mortality is very low, fitness increases as the level of care decreases; this pattern occurs because there are minimal benefits of care when offspring survive well in the absence of care. When baseline egg mortality is high, fitness increases as the level of care increases, as these are the conditions under which offspring need care the most. The findings that fitness increases as baseline egg mortality decreases and that fitness increases as the level of care increases when baseline egg mortality is high are consistent with the results presented in Figures 1 and 5. However, the results in the current figure highlight that lower levels of care will be selected for if offspring survive well without care if there are no preferences for care.

**FIGURE A4:**
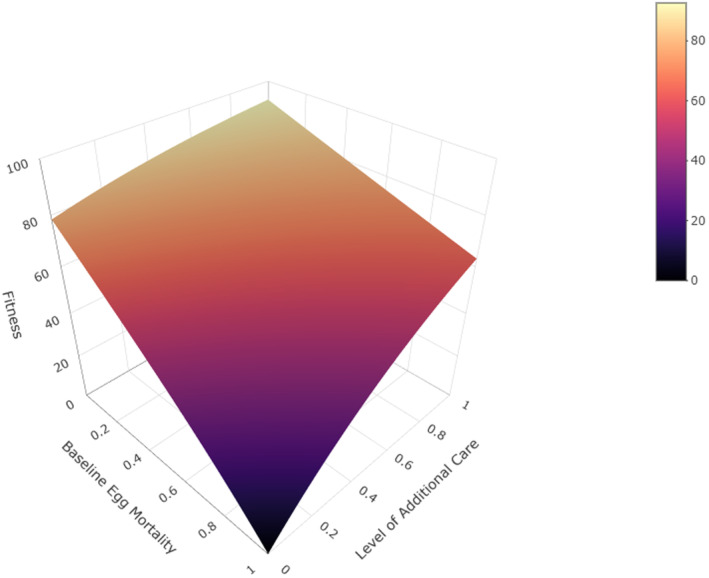
Scenario 4: The relationship between baseline egg mortality, level of care, and fitness. For Scenario 4 (mate preference for care and the mating trait), fitness increases as baseline egg mortality decreases and as the level of care increases. These findings are consistent with the results presented in Figures 1 and 5 and suggest that mate preferences for care can strongly select for parental care.

**FIGURE A5:**
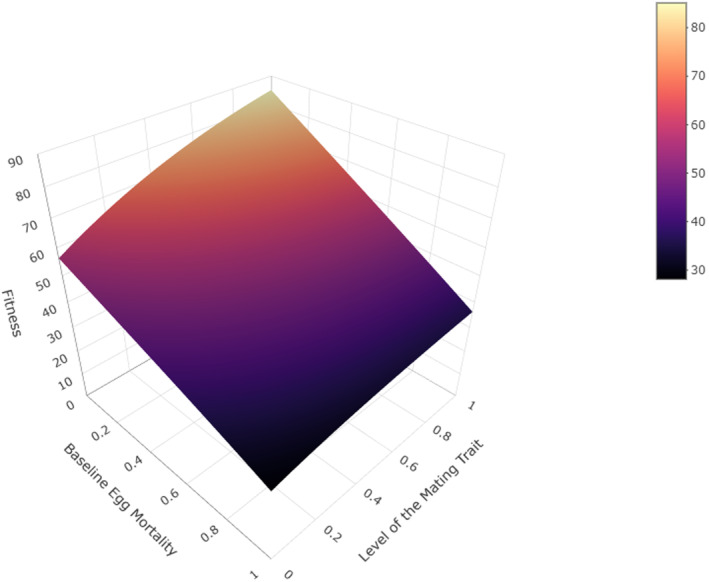
Scenario 3: The relationship between baseline egg mortality, level of the mating trait, and fitness. For Scenario 3 (mate preference for the mating trait), fitness increases as baseline egg mortality decreases and as the level of the mating trait increases. These findings are consistent with the results presented in Figures 1 and 6 and suggest that selection can strongly favor mate preferences for a non‐care trait.

**FIGURE A6:**
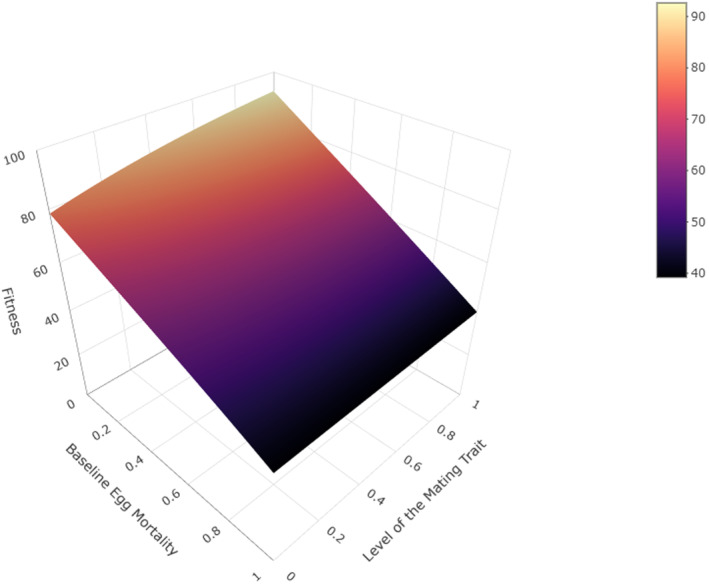
Scenario 4: The relationship between baseline egg mortality, level of the mating trait, and fitness. For Scenario 4 (mate preference for care and the mating trait), fitness increases as baseline egg mortality decreases and as the level of the mating trait increases. These findings are consistent with the results presented in Figures 1 and 6 and suggest that selection can strongly favor mate preferences for a non‐care trait.

When mate preferences for care are absent (Scenarios 1 and 3), the qualitative relationship between fitness and the level of additional care will depend on baseline egg mortality (Figures A1 and A3). When baseline egg mortality is high and no mate preferences exist, fitness increases as the level of additional care increases, as these are the conditions under which offspring need care the most (Figures A1 and A3). In contrast, if baseline egg mortality is very low, fitness will decrease as the level of additional care increases when there are no mate preferences for care (Figures A1 and A3). This pattern occurs because the benefits of care are minimal or absent when offspring survive well without additional care if there are no mate preferences for care. However, if a mate preference for additional care exists (Scenarios 2 and 4), fitness increases as the level of additional care increases across all levels of baseline egg mortality (Figures A2 and A4). Thus, mate preferences for additional care can broaden the baseline egg mortality values over which we would expect greater paternal care to occur. The qualitative relationship between fitness and the level of the mating trait does not depend on baseline egg mortality (Figures A5 and A6).

**FIGURE A7:**
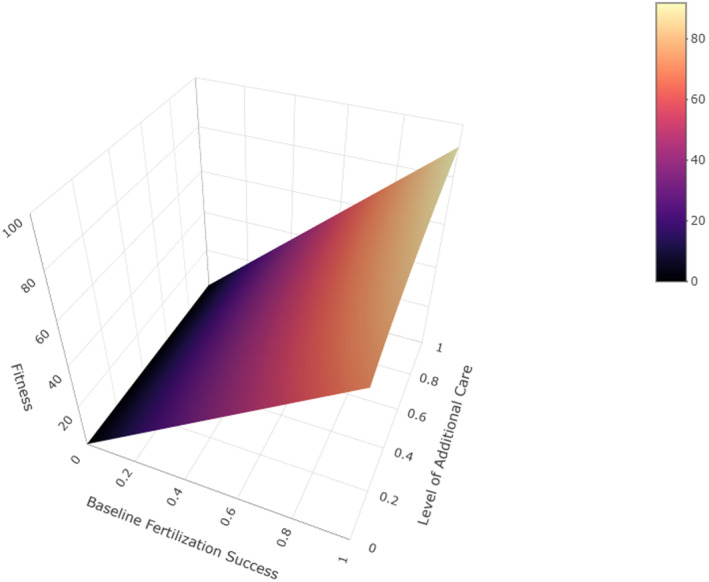
Scenario 1: The relationship between baseline fertilization success, level of care, and fitness. For Scenario 1 (no mate preferences), fitness increases as baseline fertilization success increases and as care increases. The findings are consistent with the results presented in Figures 2 and 5 and suggest that mate preferences for care can strongly select for parental care.

**FIGURE A8:**
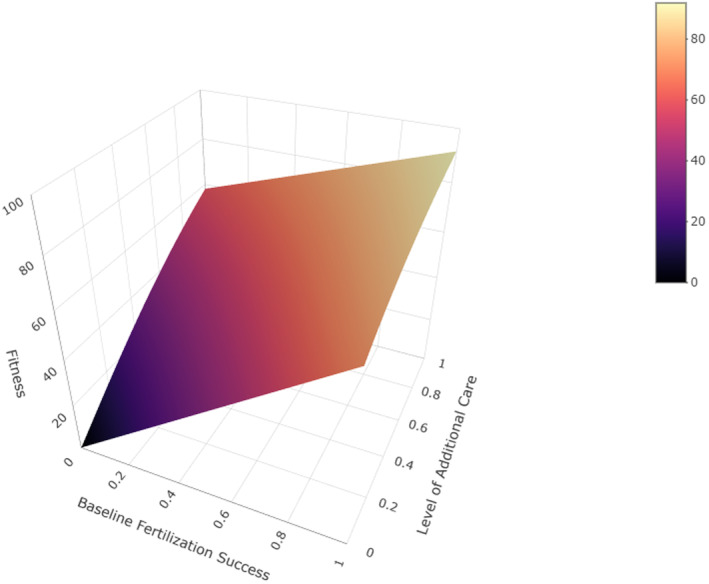
Scenario 2: The relationship between baseline fertilization success, level of care, and fitness. For Scenario 2 (mate preference for care), fitness increases as baseline fertilization success increases and as care increases. The findings are consistent with the results presented in Figures 2 and 5 and suggest that mate preferences for care can strongly select for parental care.

**FIGURE A9:**
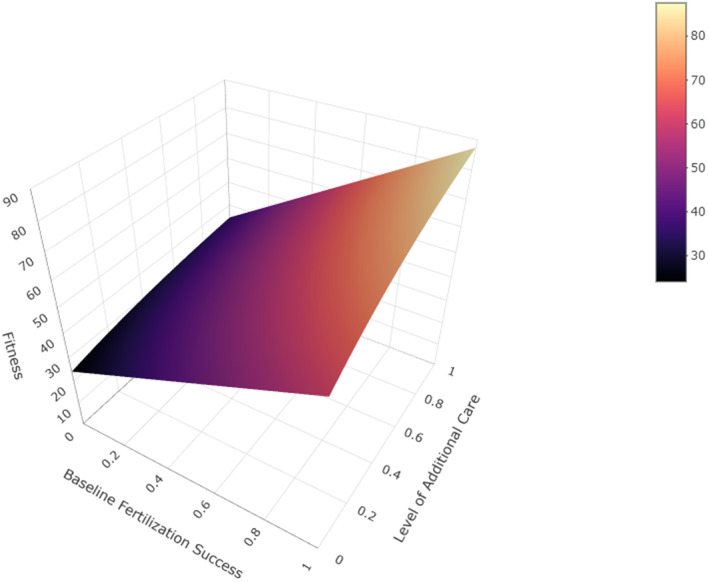
Scenario 3: The relationship between baseline fertilization success, level of care, and fitness. For Scenario 3 (mate preference for the mating trait), fitness increases as baseline fertilization success increases and as care increases. The findings are consistent with the results presented in Figures 2 and 5.

**FIGURE A10:**
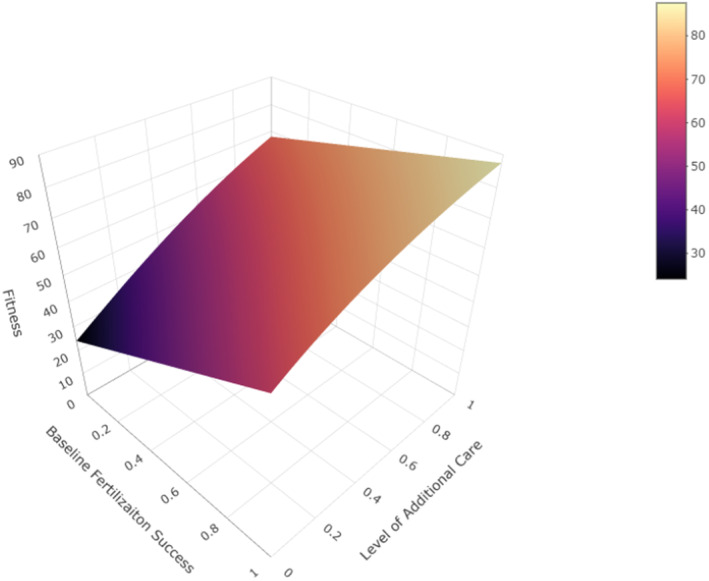
Scenario 4: The relationship between baseline fertilization success, level of care, and fitness. For Scenario 4 (mate preferences for care and the mating trait), fitness increases as baseline fertilization success increases and as care increases. The findings are consistent with the results presented in Figures 2 and 5.

**FIGURE A11:**
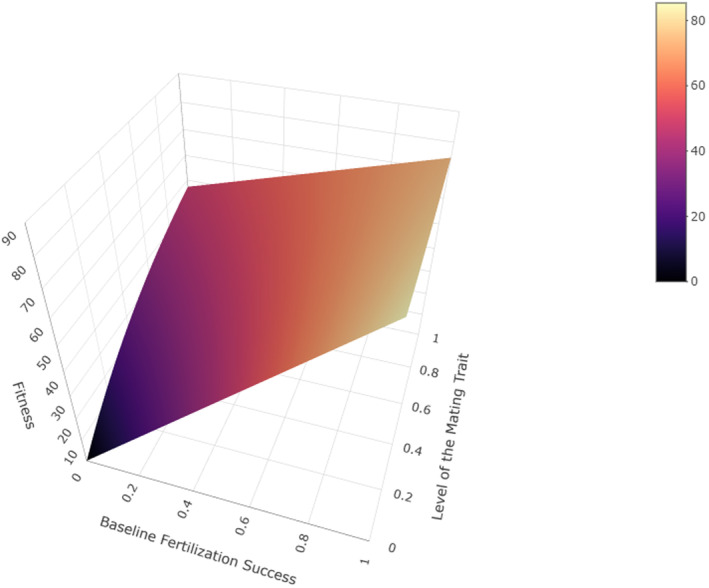
Scenario 3: The relationship between baseline fertilization success, level of the mating trait, and fitness. For Scenario 3 (mate preference for the mating trait), fitness increases as baseline fertilization success increases. When baseline fertilization success is low, fitness increases as the mating trait increases. When baseline fertilization success is high, fitness decreases as the mating trait increases; since baseline fertilization success is already high, benefits of the mating trait are minimal, which leads to greater fitness at lower levels of the mating trait.

**FIGURE A12:**
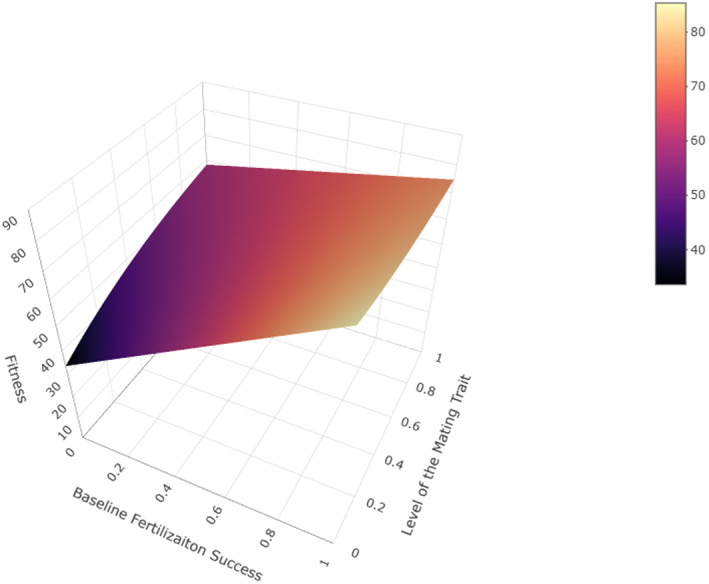
Scenario 4: The relationship between baseline fertilization success, level of the mating trait, and fitness. For Scenario 4 (mate preferences for care and the mating trait), fitness increases as baseline fertilization success increases. When baseline fertilization success is low, fitness increases as the mating trait increases. When baseline fertilization success is high, fitness decreases as the mating trait increases; since baseline fertilization success is already high, benefits of the mating trait are minimal, which leads to greater fitness at lower levels of the mating trait.

The qualitative relationship between fitness and the level of additional care does not depend on baseline fertilization success (Figures A7, A8, A9, A10). However, the qualitative relationship between fitness and the level of the mating trait depends on baseline fertilization success (Figures A11, A12). When baseline fertilization success is low, fitness increases as the mating trait increases. When baseline fertilization success is high, fitness decreases as the mating trait increases; since baseline fertilization success is already high, benefits of the mating trait are minimal, which leads to greater fitness at lower levels of the mating trait (Figures A11, A12).

**FIGURE A13:**
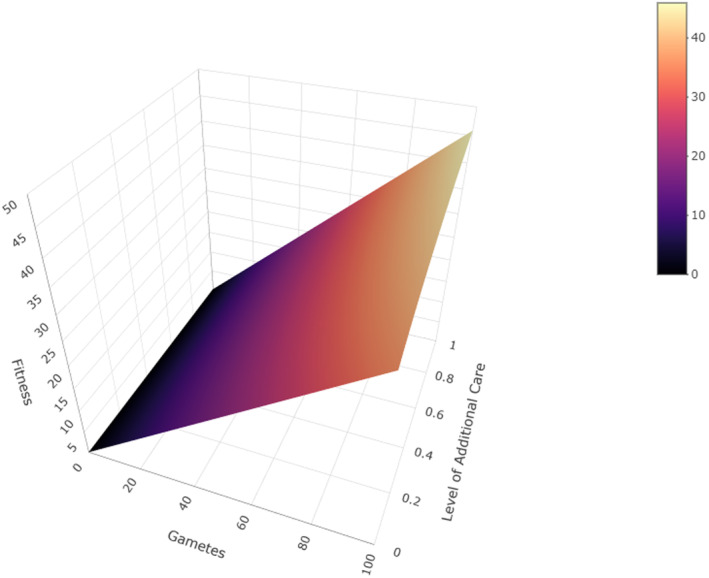
Scenario 1: The relationship between the number of available gametes, level of care, and fitness. For Scenario 1 (no mate preferences), fitness increases as the number of reproductively available gametes increases and the level of parental care increases. The findings are consistent with the results presented in Figures 3 and 5.

**FIGURE A14:**
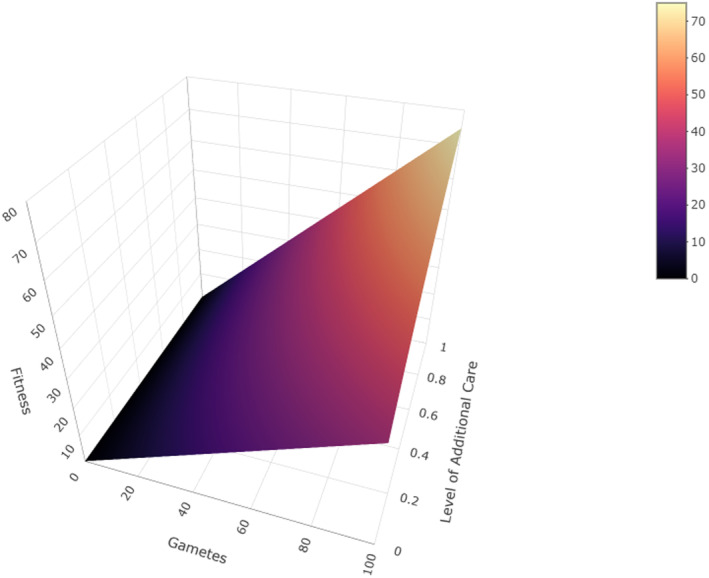
Scenario 2: The relationship between the number of available gametes, level of care, and fitness. For Scenario 2 (mate preference for care), fitness increases as the number of reproductively available gametes increases and the level of parental care increases. The findings are consistent with the results presented in Figures 3 and 5.

**FIGURE A15:**
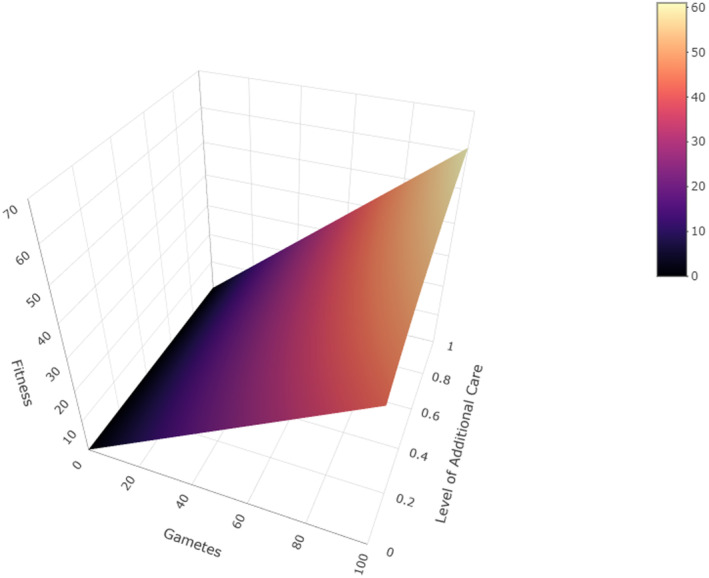
Scenario 3: The relationship between the number of available gametes, level of care, and fitness. For Scenario 3 (mate preference for the mating trait), fitness increases as the number of reproductively available gametes increases and the level of parental care increases. The findings are consistent with the results presented in Figures 3 and 5.

**FIGURE A16:**
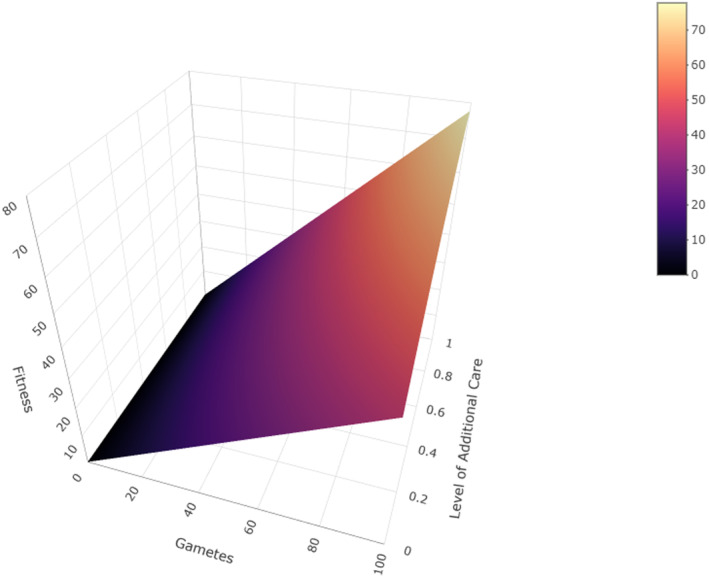
Scenario 4: The relationship between the number of available gametes, level of care, and fitness. For Scenario 4 (mate preferences for care and the mating trait), fitness increases as the number of reproductively available gametes increases and the level of parental care increases. The findings are consistent with the results presented in Figures 3 and 5.

**FIGURE A17:**
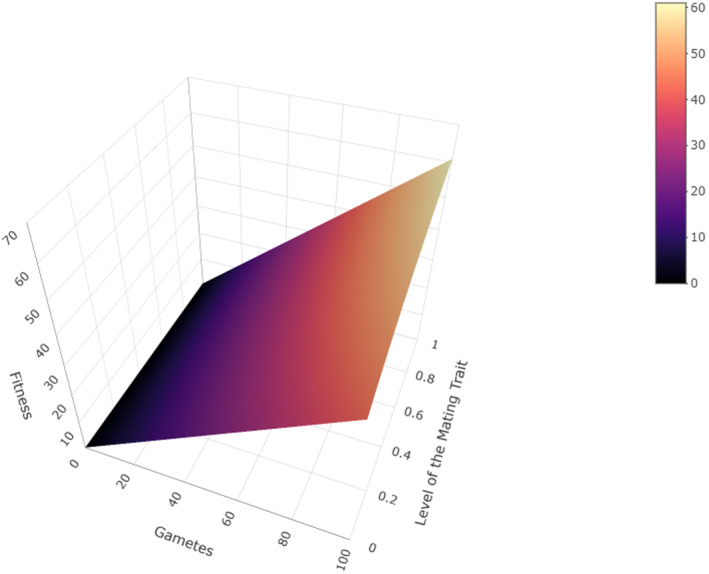
Scenario 3: The relationship between the number of available gametes, the level of the mating trait, and fitness. For Scenario 3 (mate preference for the mating trait), fitness increases as the number of reproductively available gametes increases and the level of parental care increases. The findings are consistent with the results presented in Figures 3 and 5.

**FIGURE A18:**
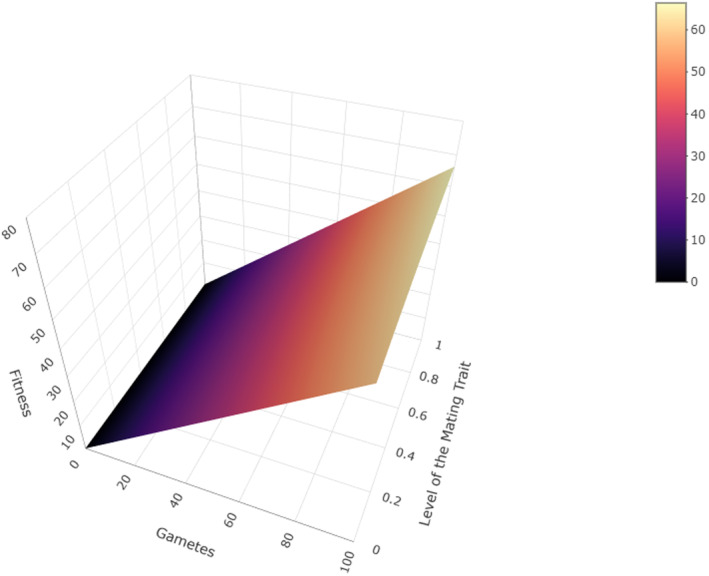
Scenario 4: The relationship between the number of available gametes, the level of the mating trait, and fitness. For Scenario 4 (mate preferences for care and the mating trait), fitness increases as the number of reproductively available gametes increases and the level of parental care increases. The findings are consistent with the results presented in Figures 3 and 5.

The qualitative relationship between fitness and the level of additional care does not depend on the number of female gametes in the population (Figures A13, A14, A15, A16). Similarly, the qualitative relationship between fitness and the level of the mating trait does not depend on the number of female gametes in the population (Figures A17, A18).

**FIGURE A19:**
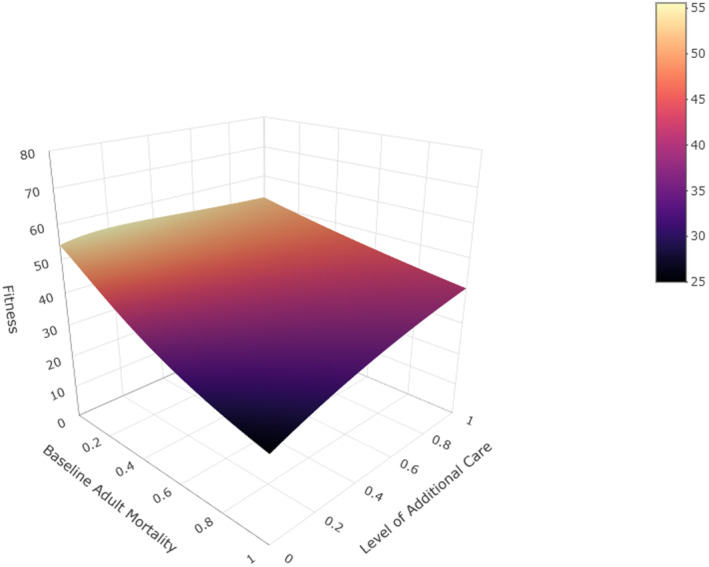
Scenario 1: The relationship between baseline adult mortality, level of care, and fitness. For Scenario 1 (no mate preferences), fitness decreases as baseline adult mortality increases. When baseline adult mortality is low, fitness increases as care decreases. When baseline adult mortality is high, fitness increases as care increases. The findings that fitness increases as baseline adult mortality decreases and that fitness, in some cases, increases as the level of care increases are consistent with the results presented in Figures 4 and 5. However, the results in the current figure highlight that the level of care selected for will depend on baseline adult mortality when mate preferences are absent.

**FIGURE A20:**
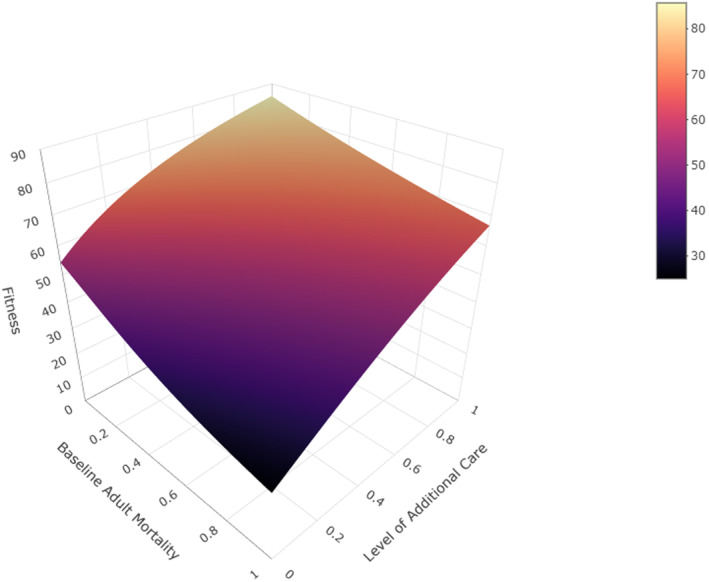
Scenario 2: The relationship between baseline adult mortality, level of care, and fitness. For Scenario 2 (mate preference for care), fitness decreases as baseline adult mortality increases and as care increases. The findings are consistent with the results presented in Figures 4 and 5 and suggest that mate preferences for care can strongly select for parental care.

**FIGURE A21:**
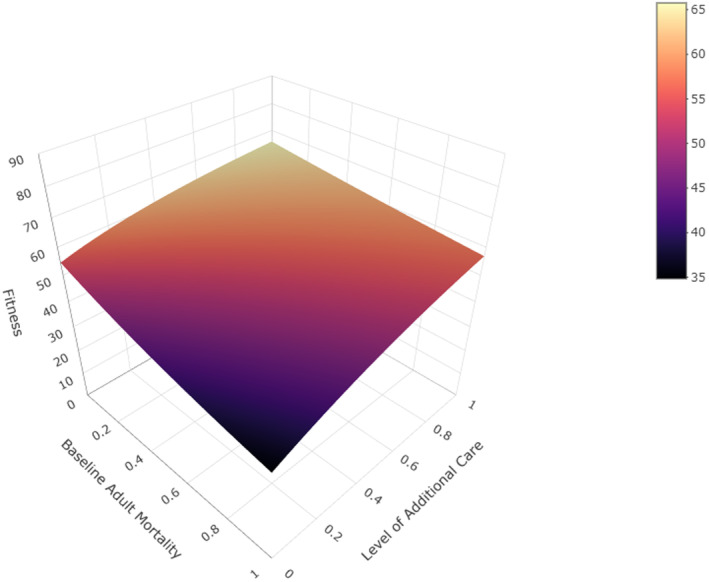
Scenario 3: The relationship between baseline adult mortality, level of care, and fitness. For Scenario 3 (mate preference for the mating trait), fitness decreases as baseline adult mortality increases and as care increases. The findings are consistent with the results presented in Figures 4 and 5 and suggest that mate preferences for a non‐care mating trait can lead to conditions that strongly select for parental care.

**FIGURE A22:**
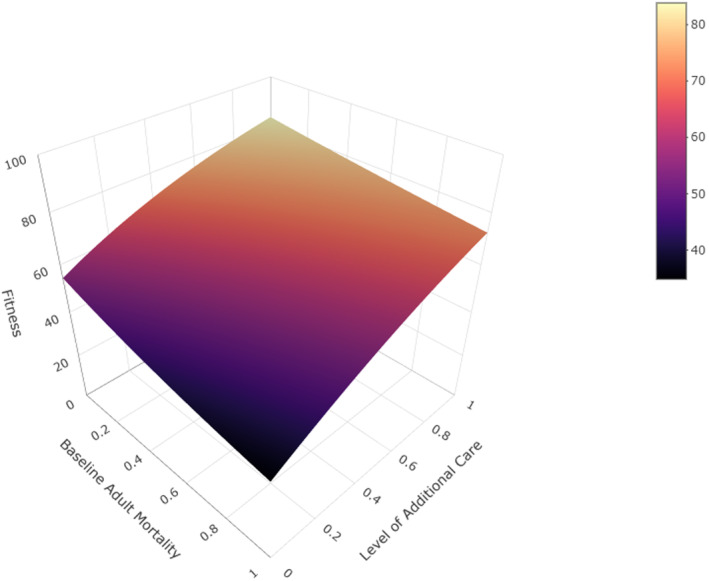
Scenario 4: The relationship between baseline adult mortality, level of care, and fitness. For Scenario 4 (mate preference for care and the mating trait), fitness decreases as baseline adult mortality increases and as care increases. The findings are consistent with the results presented in Figures 4 and 5 and suggest that mate preferences for care and a non‐care mating trait can lead to conditions that strongly select for parental care.

**FIGURE A23:**
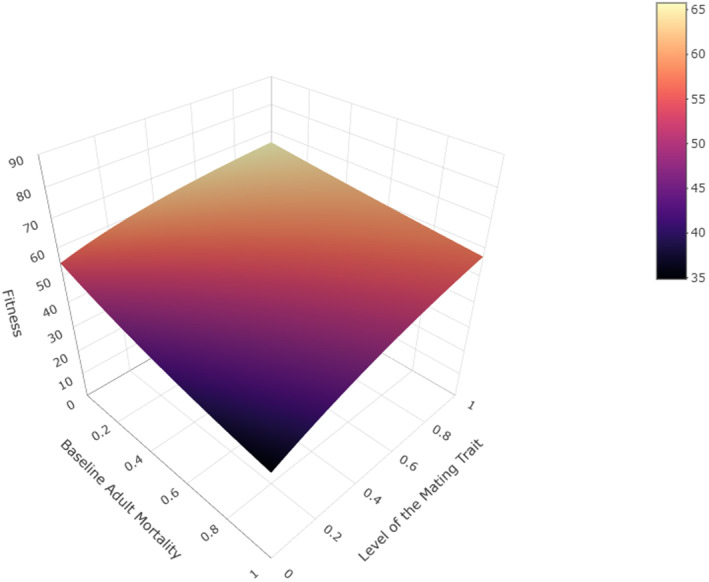
Scenario 3: The relationship between baseline adult mortality, level of the mating trait, and fitness. For Scenario 3 (mate preference for the mating trait), fitness decreases as baseline adult mortality increases and as the mating trait increases. The findings are consistent with the results presented in Figures 4 and 6.

**FIGURE A24:**
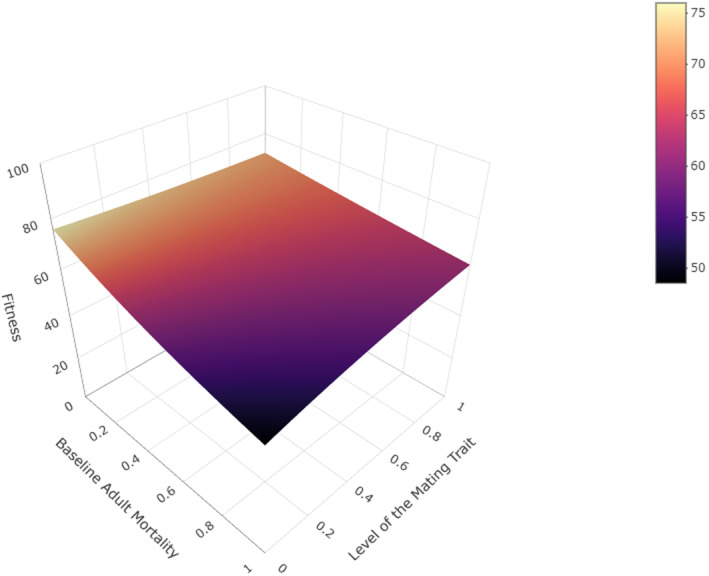
Scenario 4: The relationship between baseline adult mortality, level of the mating trait, and fitness. For Scenario 4 (mate preferences for care and the mating trait), fitness decreases as baseline adult mortality increases. When baseline adult mortality is low, fitness decreases as the mating trait increases. When baseline adult mortality is high, fitness increases as the mating trait increases. The findings are partially consistent with the results presented in Figures 4 and 6 but further highlight that life history and mate preferences can interact to influence selection on a mating trait.

When mate preferences are absent, the qualitative relationship between fitness and additional care will depend on baseline adult male mortality (Figure A19). When baseline adult male mortality is low, fitness increases as additional care decreases (Figure A19). In contrast, when baseline adult mortality is high, fitness increases as additional care increases (Figure A19). However, when mate preferences exist for care and/or the mating trait, there will be a positive relationship between additional care and fitness across the full range of baseline adult mortality levels (Figures A20, A21, A22), suggesting that mate preferences for additional care and/or another trait can broaden the range of life‐history parameters over which more paternal care is favored. When there is only a preference for the mating trait, the qualitative relationship between fitness and the mating trait will not depend on baseline adult mortality (Figure A23). In contrast, when there is a preference for both additional care and the mating trait, the qualitative relationship between fitness and the mating trait depends on baseline adult mortality (Figure A24). When baseline adult mortality is low, fitness decreases as the mating trait increases. When baseline adult mortality is high, fitness increases as the mating trait increases (Figure A24). These results suggest that mate preferences can alter the life‐history parameter values under which additional care and/or another trait will be most strongly selected for.

## 4 DISCUSSION

Here, we investigated how life history and female mate preferences interact to influence the fitness associated with investment in paternal care and a preferred male mating trait. We show how offspring mortality, fertilization success, and adult male mortality are expected to have strong influences on the conditions under which sexual selection will favor investment in additional male care versus a preferred mating trait. Our finding that sexual selection can favor additional male care is consistent with previous theoretical (Alonzo, 2012; Stiver & Alonzo, 2009) and empirical research (Forsgren, 1997; Künzler & Bakker, 2000; Lindström et al., 2006; Requena et al., 2013; Tallamy, 2000) that suggests paternal care is influenced by mate preferences. However, the finding that life history will influence the conditions under which additional male care versus another male trait will be favored by sexual selection is, to the best of our knowledge, novel. Indeed, our results provide an initial look at the life‐history conditions under which additional male care versus a non‐care mating trait is most likely to be favored evolutionarily. Such research will broaden our understanding of sexual selection on male traits.

When male investment in additional care and a mating trait is constrained to a fixed value and egg mortality is low due to high levels of baseline male care, investing in additional care or in both additional care and a mating trait will be associated with high fitness when additional care and/or the mating trait are preferred by females in mate choice. That is, when eggs survive well, sexual selection can favor either additional paternal care or a non‐care mating trait. In contrast, when male investment in additional care and a mating trait is constrained to a fixed value and egg mortality is high due to low baseline levels of male care, investing in a mating trait is likely to be selected against in favor of additional investment in paternal care. Specifically, at high levels of egg mortality (i.e., low baseline male care), fitness is greatest when males invest in additional care, regardless of whether additional care is preferred in mate choice or not. This suggests that high need for additional paternal care (i.e., high offspring mortality) can inhibit sexual selection for a non‐care mating trait when there is a tradeoff between investing in additional care versus a mating trait. If offspring need more care, male fitness will be greatest when males invest in additional care at the expense of investing in a sexually selected non‐care trait. When total male investment in additional care and a mating trait is not constrained to a fixed value, baseline egg mortality (i.e., baseline male care) will not influence the strategy that is associated with the greatest fitness. Under such conditions, male fitness will be relatively high when males invest in both additional care and a mating trait. However, if only one trait is preferred in mate choice, male fitness will be greatest if that trait is additional parental care. Interestingly, mate preferences for additional care can broaden the baseline egg mortality levels across which we would expect additional care to be favored. When mate preferences are absent, fitness increases as the level of additional care increases when baseline egg mortality is high (i.e., when baseline male care is low) but decreases as the level of additional care increases if baseline egg mortality is very low (i.e., if baseline male care is very high). In contrast, if additional care is preferred in mate choice, fitness increases as the level of additional care increases across all levels of baseline egg mortality (i.e., all levels of baseline male care). This suggests that the elaboration of male care can occur across a relatively broad range of egg mortalities when females prefer paternal care.

The finding that higher egg mortality will select for greater investment in additional paternal care is consistent with previous theoretical (Klug et al., 2013a, 2013b; Klug & Bonsall, 2010) and some empirical research (e.g., (Steinhart et al., 2008)). For example, in the smallmouth bass (*Micropterus dolomieu*), males provided more parental care, as evidenced by a lower likelihood of offspring abandonment, when offspring daily survival was low (Steinhart et al., 2008). Our finding that offspring mortality can interact with sexual selection to influence the fitness associated with paternal care is, to the best of our knowledge, novel. Similarly, the finding that female mate preferences for care can broaden the baseline egg mortality levels across which we would expect additional care to be selected for is also novel.

Fertilization success influences whether sexual selection is most likely to favor additional paternal care or another male trait that is preferred in mate choice. Regardless of whether total male investment in additional care and a mating trait is constrained or not constrained to a fixed value (i.e., regardless of whether investment in additional care and a preferred mating trait trade off), investing solely in additional care, even when care is not preferred in mate choice, will result in the greatest male fitness when baseline fertilization success is high. This pattern occurs as the benefit of investing in a preferred trait is low when males already have high fertilization success. When total male investment in additional care and a mating trait is not constrained to a fixed value (i.e., when investment in additional care and a mating trait do not trade off), selection will most strongly favor investment in both additional care and a mating trait at low levels of baseline fertilization success. In contrast, when total male investment into additional care and a mating trait is constrained to a fixed value (i.e., when investment in additional care and a mating trait trade off), selection will most strongly favor investment in only additional care at low levels of baseline fertilization success. This pattern occurs as (1) investment in preferred traits will result in relatively high fitness benefits when male fertilization success is low in the absence of such investment and (2) males should invest solely in care when it is preferred if there is a tradeoff between care and a non‐care mating trait. In general, these results suggest that tradeoffs can interact with the level of baseline fertilization success to influence sexual selection for additional paternal care or another male trait. Indeed, previous theoretical research has shown that mating dynamics can influence parental care (Araujo & Moura, 2023; Azad et al., 2022; Fromhage et al., 2007; Fromhage & Jennions, 2016; Kokko & Jennions, 2008; Requena & Alonzo, 2017). Our finding that fertilization success can interact with mate preferences to influence the fitness associated with additional investment in paternal care provides further evidence of the complex relationship between mating, life history, and the evolutionary dynamics of male care.

Baseline adult male mortality also influences whether selection is most likely to favor additional paternal care or another male trait. When investment in traits is not constrained to a fixed value and baseline adult male mortality is low, fitness will be greatest when males invest in additional care if additional care is preferred in mate choice. At moderate and high values of baseline adult mortality, it becomes increasingly beneficial to invest in both additional care and the mating trait when preferences for both exist. Such a pattern likely occurs because males have reduced future reproductive potential when baseline adult mortality is high. Under such conditions, classic life‐history theory predicts that males will invest more in current reproduction (Stearns, 1992), and in our model, males increase their current reproductive success by investing in preferred traits. Similarly, other theoretical work has found that some form of parental care is more likely to originate when adult mortality is high, a life‐history condition that would be expected to be associated with short‐lived species (Klug & Bonsall, 2010).

Mate preferences for additional care and/or a mating trait can broaden the adult male mortality values over which we would expect additional paternal care to be favored. When mate preferences are absent, fitness increases as additional care decreases if baseline adult male mortality is low, whereas fitness increases as additional care increases if baseline adult mortality is high. In contrast, if females prefer additional care and/or a mating trait, fitness will increase as additional care increases across all levels of baseline adult male mortality, suggesting that mate preferences can broaden the life‐history conditions under which additional paternal care will be selected for. This finding is, to the best of our knowledge, novel and could help explain the prevalence and exaggeration of paternal care in some systems (e.g., Lindström et al., 2006; Pampoulie et al., 2004).

Empirically, paternal care is affected by adult mortality in some species. For example, in smallmouth bass males were less likely to abandon broods when daily adult survival was low (Steinhart et al., 2008). When total trait investment is constrained to a fixed value, baseline adult mortality does not influence the scenario that is associated with the greatest fitness in our model. Under these conditions, males will have the greatest fitness by investing solely in additional care if additional care is preferred in mate choice.

Under the conditions of our model, the number of reproductively available female gametes did not influence the relative fitness of the scenarios considered. However, in the current model, we did not impose a tradeoff between offspring number and size or quality, and such a tradeoff might lead to different results, particularly since offspring size is likely to affect offspring survival (Smith & Fretwell, 1974). Exploring how a tradeoff between offspring size and number influences the life‐history conditions under which additional male care is favored would be an interesting avenue of future research.

In summary, the finding that additional male care can be favored via mate preferences is consistent with previous theoretical (Alonzo, 2012; Stiver & Alonzo, 2009) and empirical research (Forsgren, 1997; Künzler & Bakker, 2000; Lindström et al., 2006; Pampoulie et al., 2004; Requena et al., 2013; Tallamy, 2000). Likewise, the finding that life history influences the evolution of paternal care is consistent with earlier theoretical research (Klug et al., 2013a, 2013b). However, the general finding that basic life history and sexual selection can interact to influence the fitness associated with additional paternal care and/or a non‐care mating trait is novel. Our results provide a range of testable predictions of when we would expect the elaboration of paternal care versus investment in a non‐care preferred mating trait.

Importantly, our aim with this work was to develop a fairly straightforward model to explore how life history can interact with mate preference scenarios to influence male investment strategies. While the current work provides a first look at how life history and sexual selection can interact to influence the fitness associated with investment in additional male care versus another preferred trait, we did not consider a range of important factors, including co‐evolution between male and female traits, adaptive dynamics, frequency dependence, etc. Such factors are known to influence the evolution of parental investment and sexually selected traits (e.g., Alonzo, 2010; Araujo & Moura, 2023; Azad et al., 2022; Fromhage et al., 2007; Requena et al., 2013; Stiver & Alonzo, 2009). Thus, in future work, it will be important to consider how such complexities (co‐evolution, frequency dependence, adaptive dynamics, etc.) might alter the predictions of our model. In the future, it will also be interesting to explore empirically the links between paternal care level, mate preferences, and life‐history traits including baseline egg mortality, baseline adult male mortality, and reproductive success.

## AUTHOR CONTRIBUTIONS

**Taya de Blonk:** Conceptualization (equal); formal analysis (lead); investigation (lead); software (lead); visualization (lead); writing – original draft (equal); writing – review and editing (supporting). **Isimeme N. Udu:** Investigation (supporting); methodology (supporting); software (supporting). **Michael B. Bonsall:** Conceptualization (equal); investigation (supporting); methodology (lead); software (supporting); writing – original draft (supporting); writing – review and editing (supporting). **Hope Klug:** Conceptualization (equal); formal analysis (supporting); funding acquisition (lead); investigation (equal); methodology (equal); project administration (lead); writing – original draft (equal); writing – review and editing (equal).

## FUNDING INFORMATION

This material is based upon work supported by the National Science Foundation under grant no. DEB 1552721 (to H.K.) and an REU supplement to grant no. DEB 1552721 (to H.K. and I.N.U.).

## CONFLICT OF INTEREST STATEMENT

The authors have no competing interests.

## Supporting information


Data S1.

## Data Availability

All data are included in the figures. Scripts are available at https://osf.io/2xfb9/.

## APPENDIX A

### A.1

To determine whether life‐history parameters interact with the level of care or the level of the mating trait to influence fitness, we provide 3D plots of (1) baseline egg mortality, care, and fitness (Figures A1, A2, A3, A4), (2) baseline egg mortality, the mating trait, and fitness (Figures A5, A6), (3) baseline fertilization success, care, and fitness (Figures A7, A8, A9, A10), (4) baseline fertilization success, the mating trait, and fitness (Figures A11, A12), (5) the number of available female gametes, care, and fitness (Figures A13, A14, A15, A16), (6) the number of available female gametes, the mating trait, and fitness (Figures A17, A18), and (7) baseline adult male mortality, care, and fitness (Figures A19, A20, A21, A22), and (8) baseline adult male mortality, the mating trait, and fitness (Figures A23, A24). Scenarios and fitness functions are as described in the main body of the manuscript. Unless noted in a given figure, parameter values are as follows: *a* = 1.0, *r*_*o*_ = 0.5, *d*_*ao*_ = 0.5, *d*_*eo*_ = 0.5, *n* = 5.0, *b* = 100.
