# Supplementary material for: Sexual selection and life history interact to influence the evolution of paternal care
Source: Ecol Evol. 2024 Aug 21;14(8):e70189. doi: 10.1002/ece3.70189 (PMC11338692; doi:10.1002/ece3.70189)

Supplementary Materials

Figures S1-S14 graphically depict the trade-off functions associated with investment in additional care and a non-care mating trait. Trade-offs, Scenarios, and modelling dynamics are as described in the main text. Unless otherwise noted, in Figs. S1-S14, *r_o_=0.5, b = 100, d_Ao_=0.5, n = 5.0, d_Ao_ = 0.5, m = 0.5 or 0.25* (depending on scenario considered), *c = 0.5 or 0.25* (depending on scenario considered).

Scenario 1 - no mate preferences (S1-S2)

Figure S1. Cost of additional care: additional care increases adult death rate.


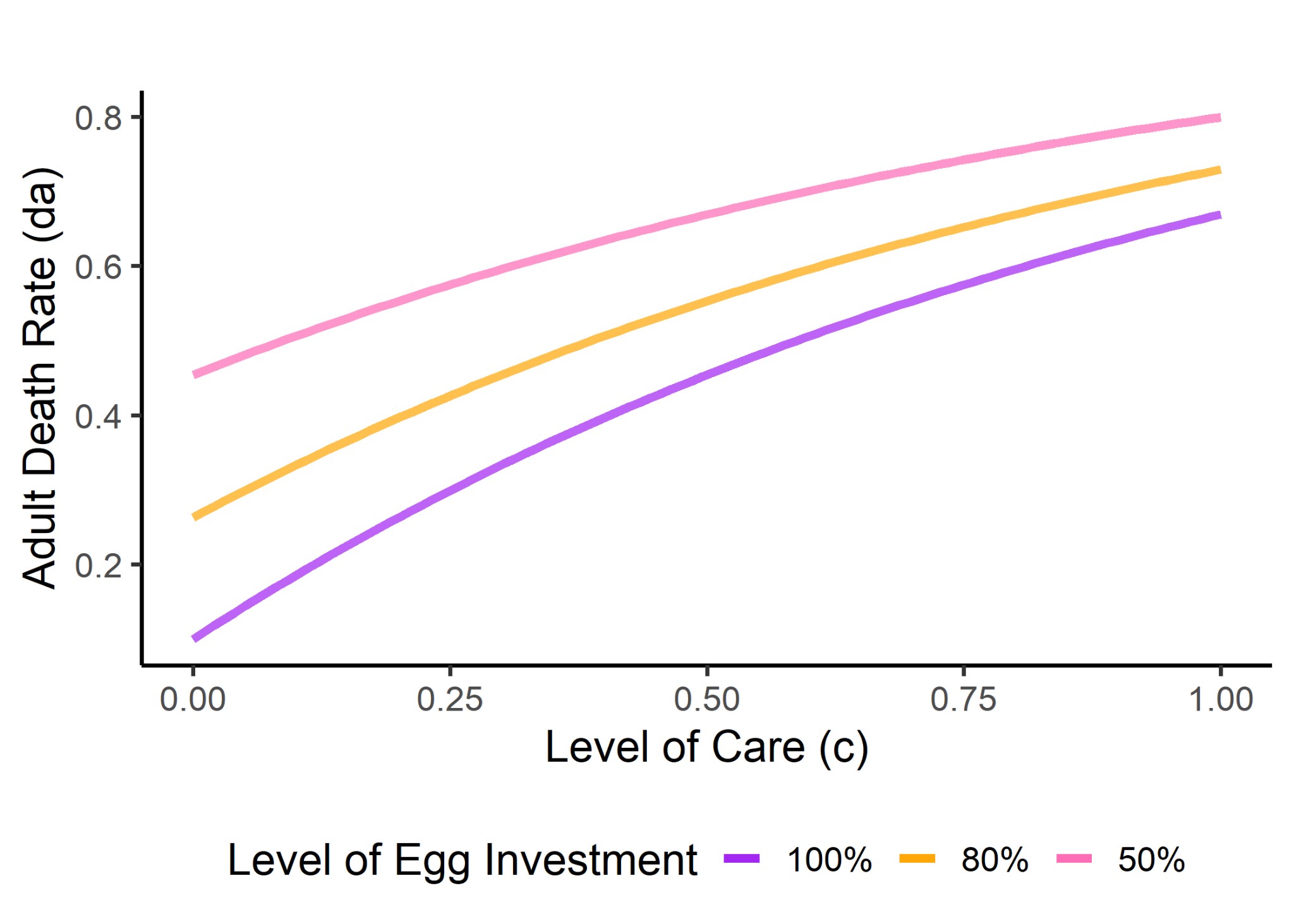


Figure S2. Benefit of additional care: additional care decreases egg death rate.


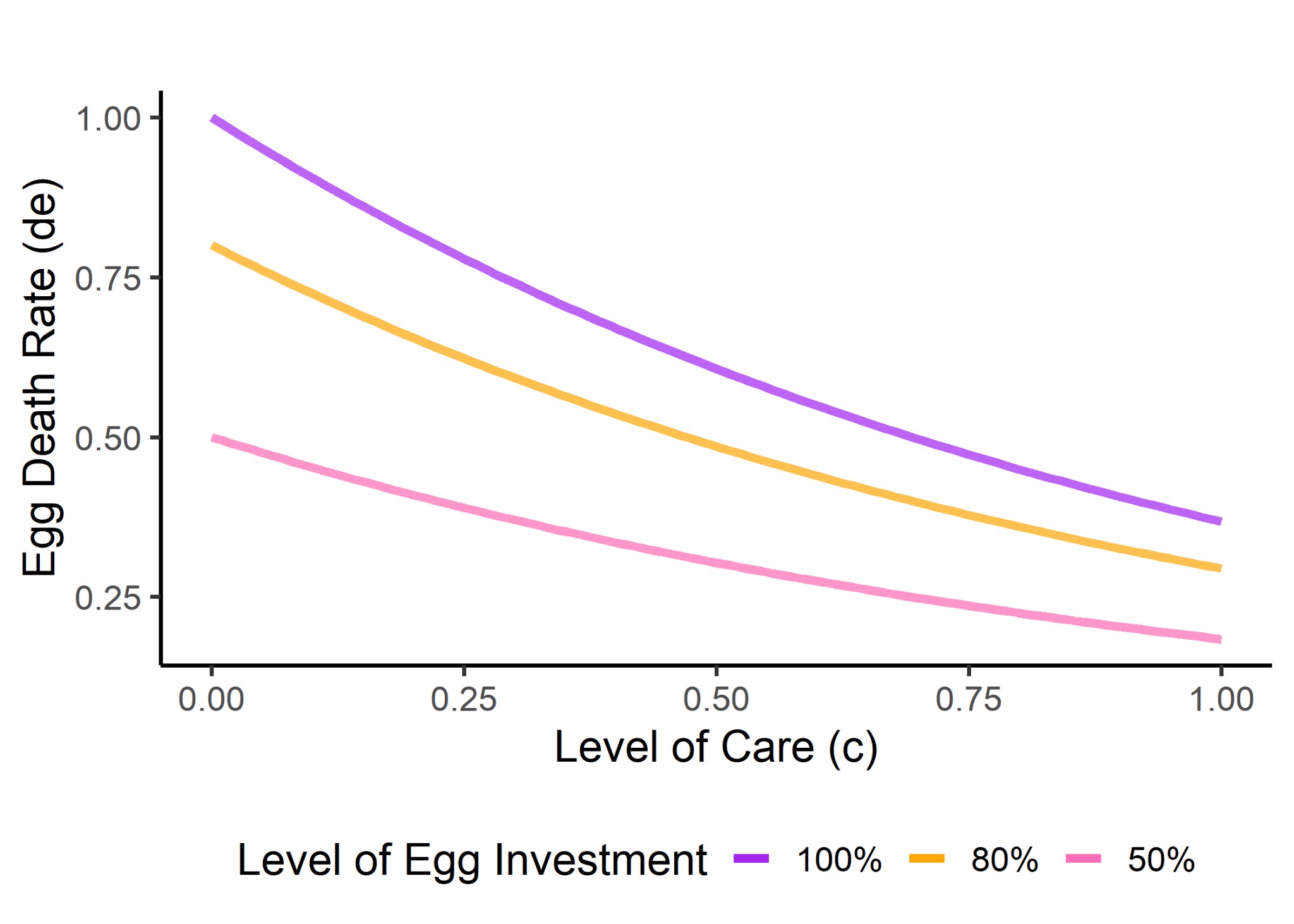


Scenario 2 - mate preference for additional care only (S3-S5)

Figure S3. Cost of additional care: additional care increases adult death rate.


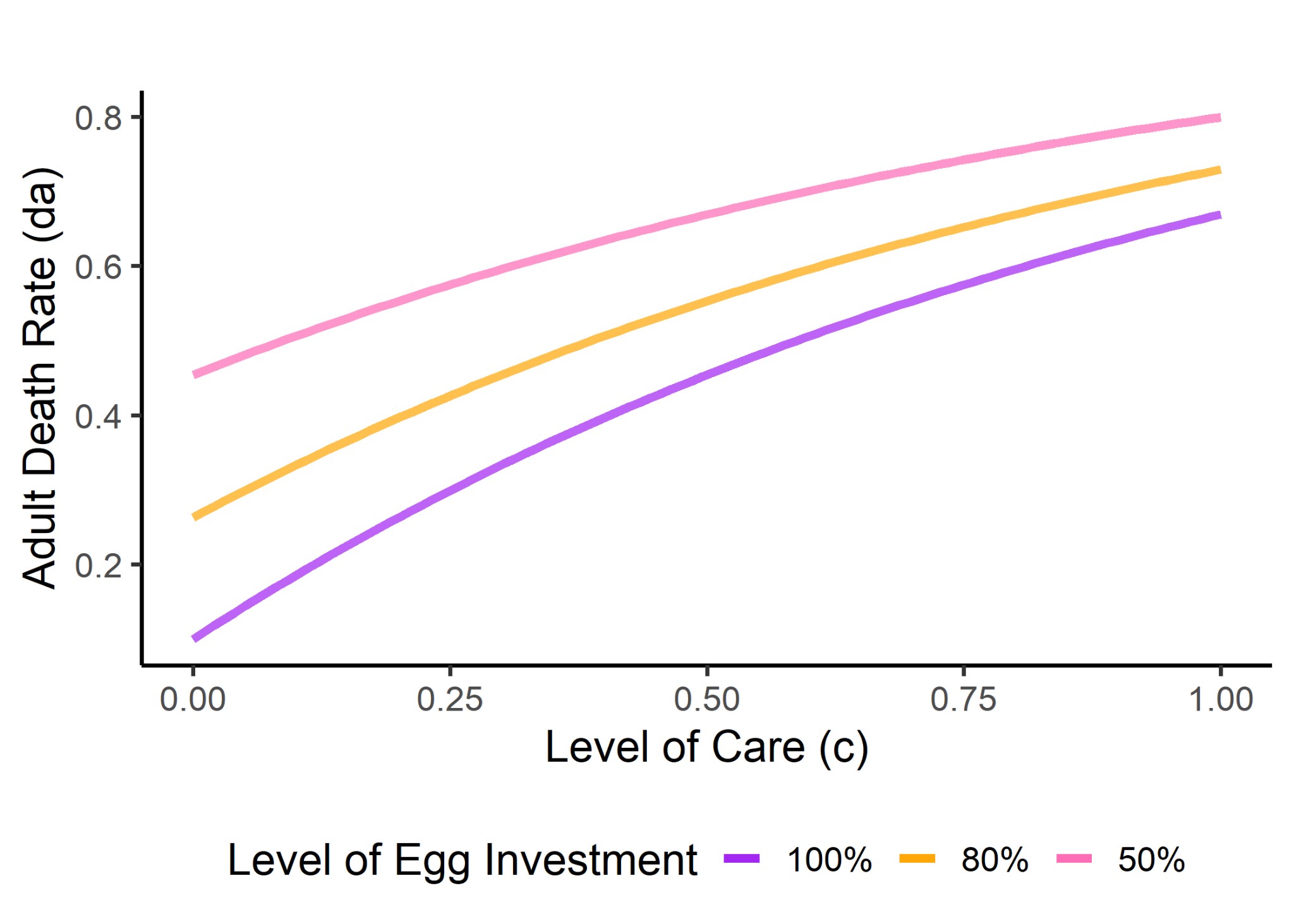


Figure S4. Benefit of additional care: additional care decreases egg death rate.


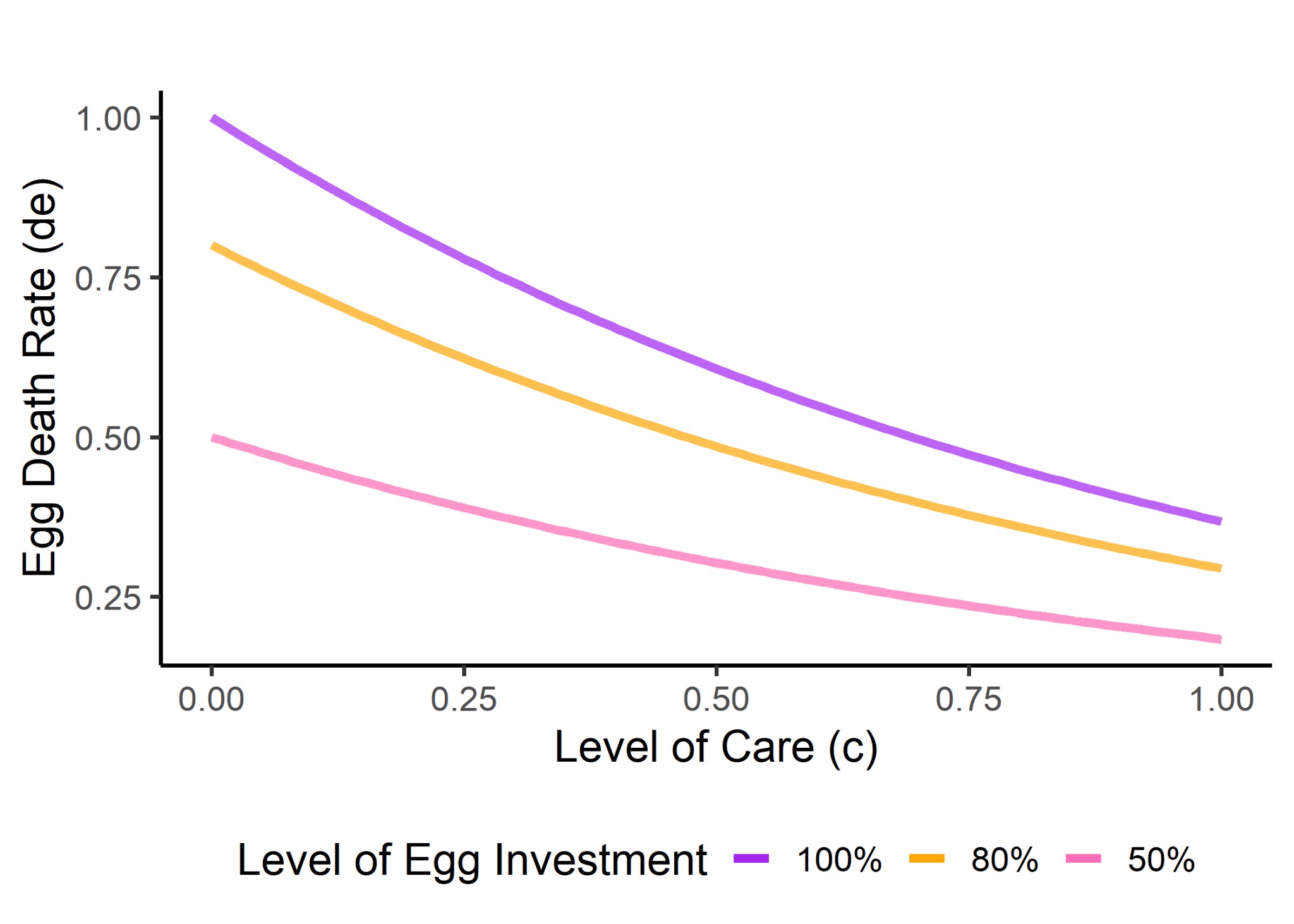


Figure S5. Benefit of additional care: additional care increases fertilization rate.


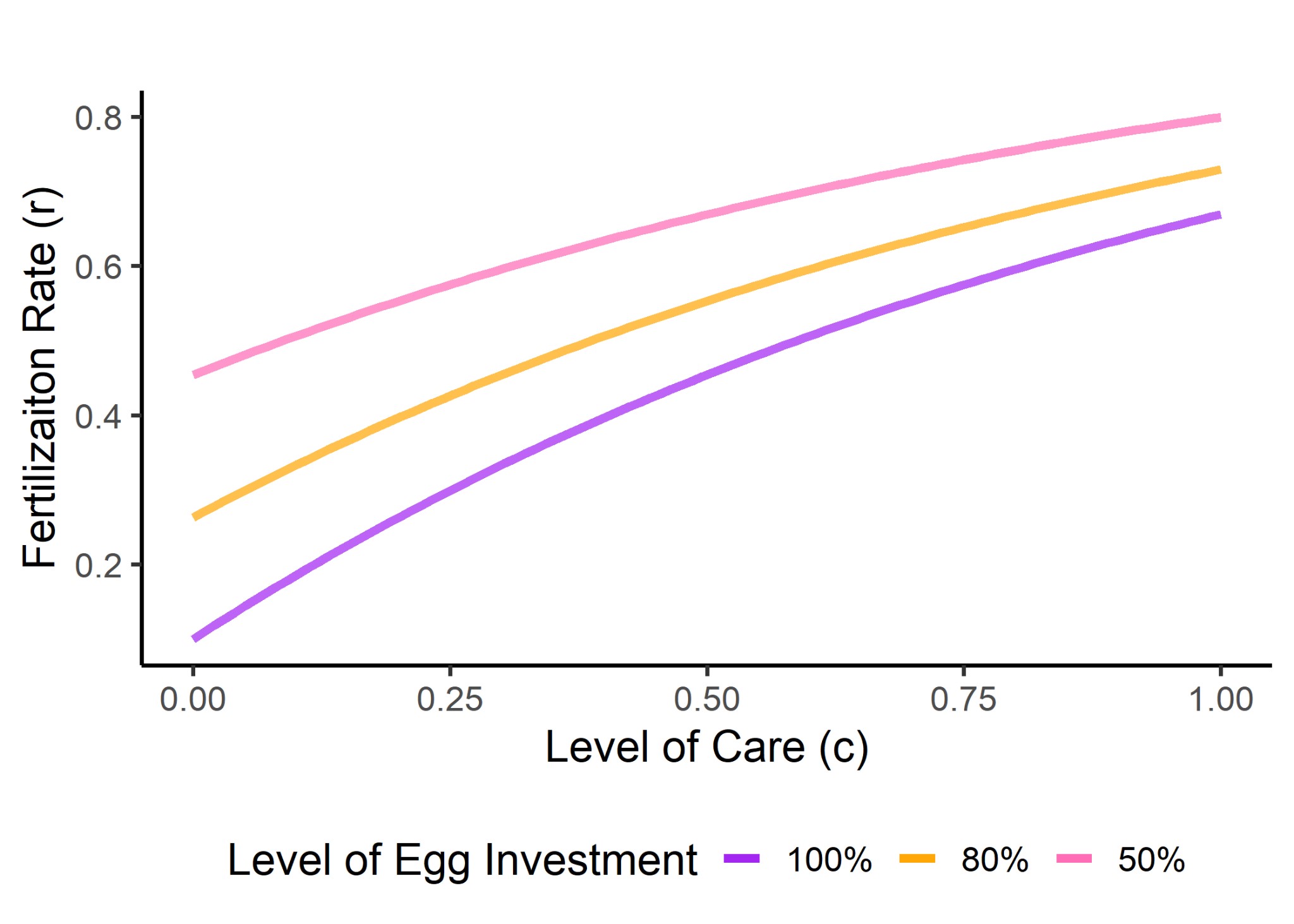


Scenario 3: Mate preference for the mating trait only (S6-S9)

Figure S6. Cost of additional care: additional care increases adult death rate.


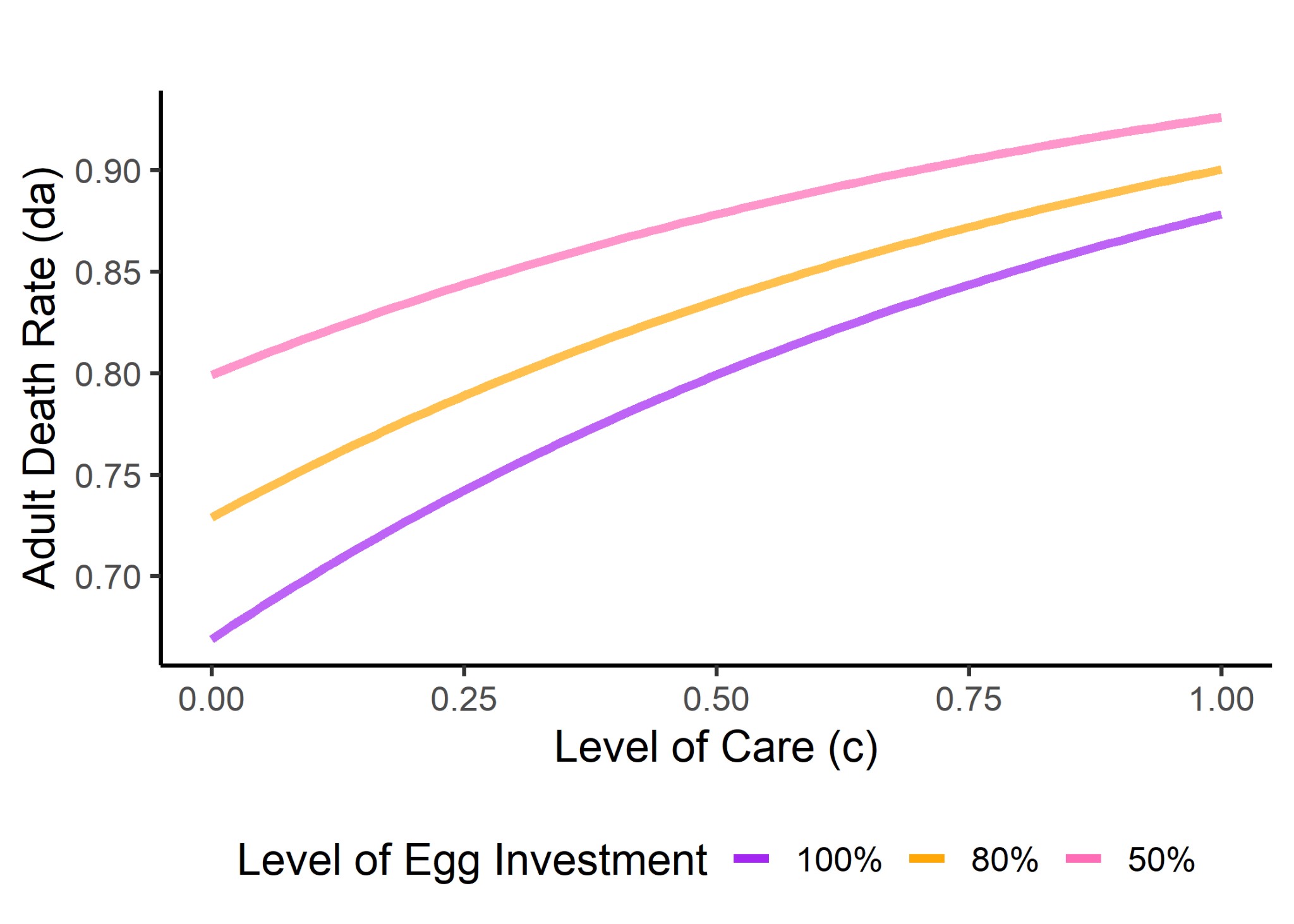


Figure S7. Benefit of additional care: additional care decreases egg death rate.


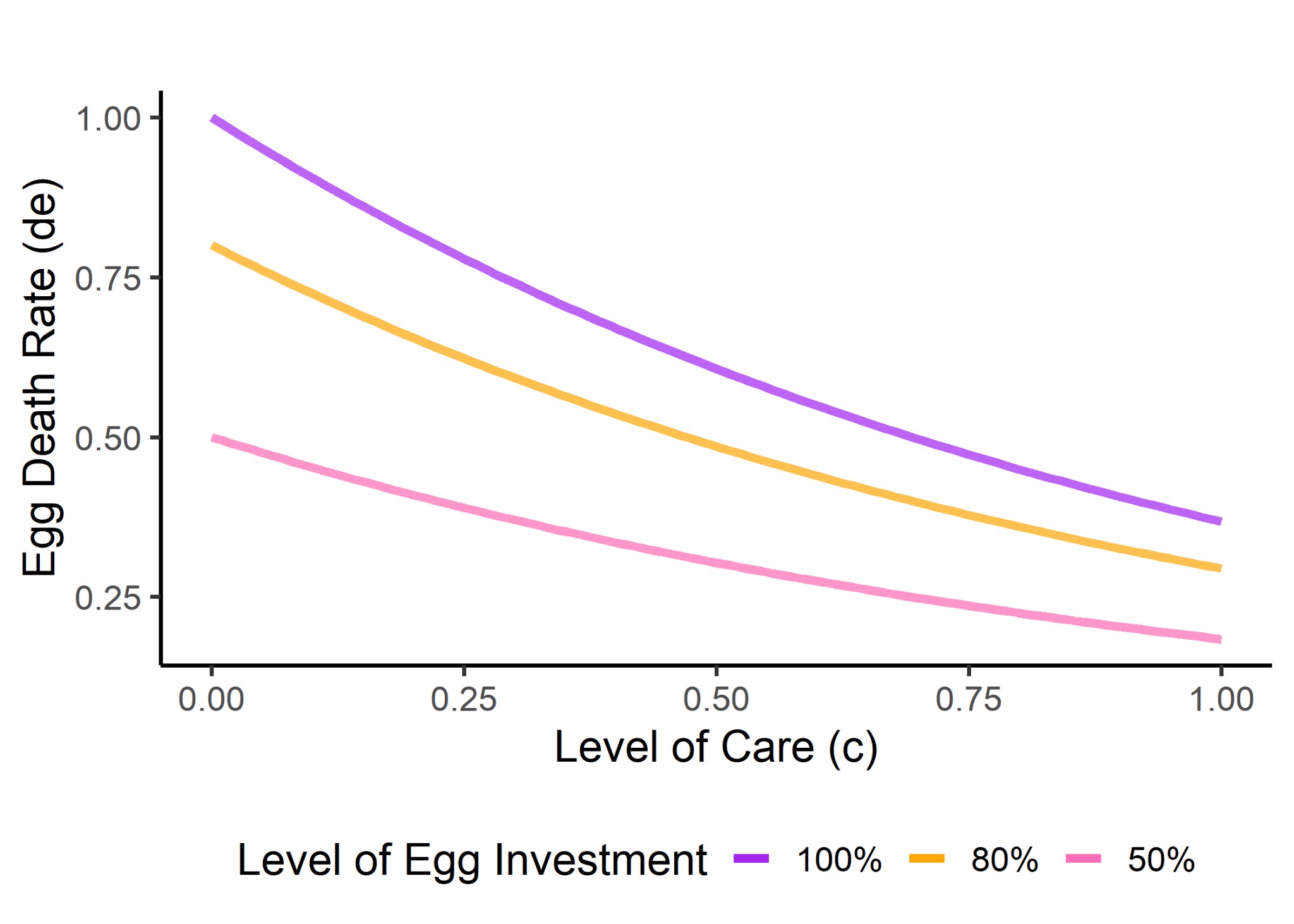


Figure S8. Cost of the mating trait: mating trait increases adult death rate.


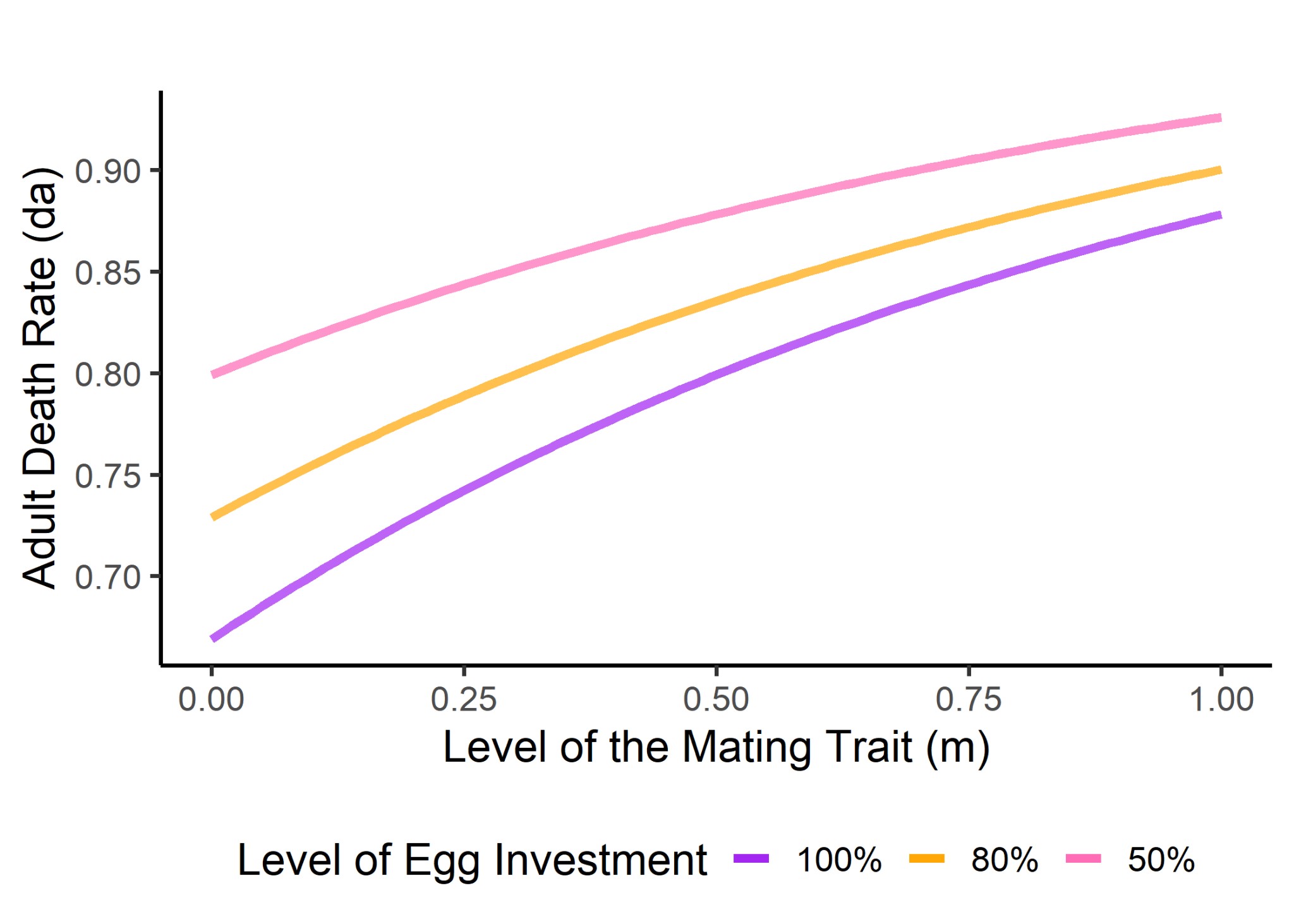


Figure S9. Benefit of the mating trait: mating trait increases fertilization rate.


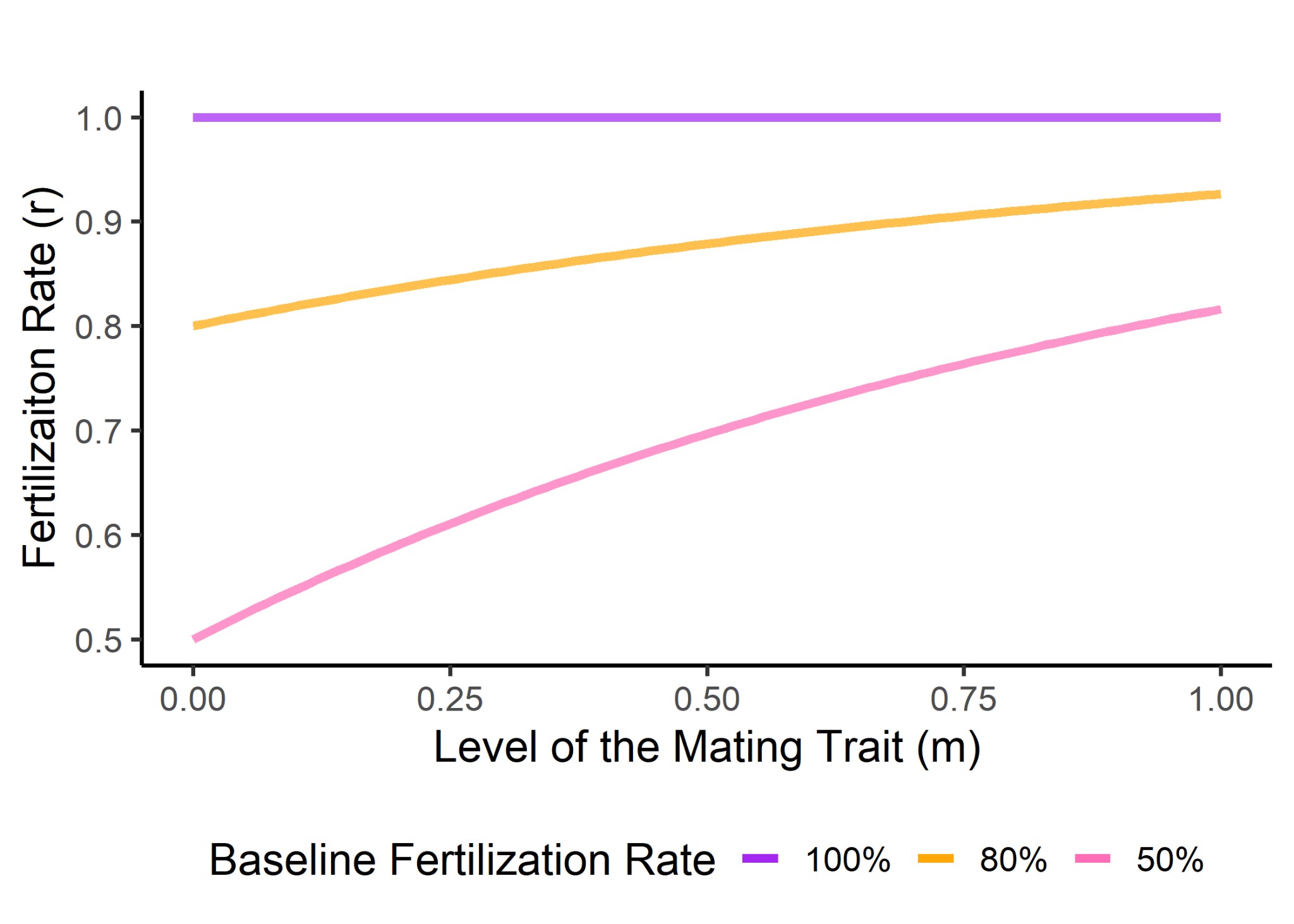


Scenario 4: Mate preference for additional care and the mating trait (S10-14)

Figure S10. Cost of additional care: additional care increases adult death rate.


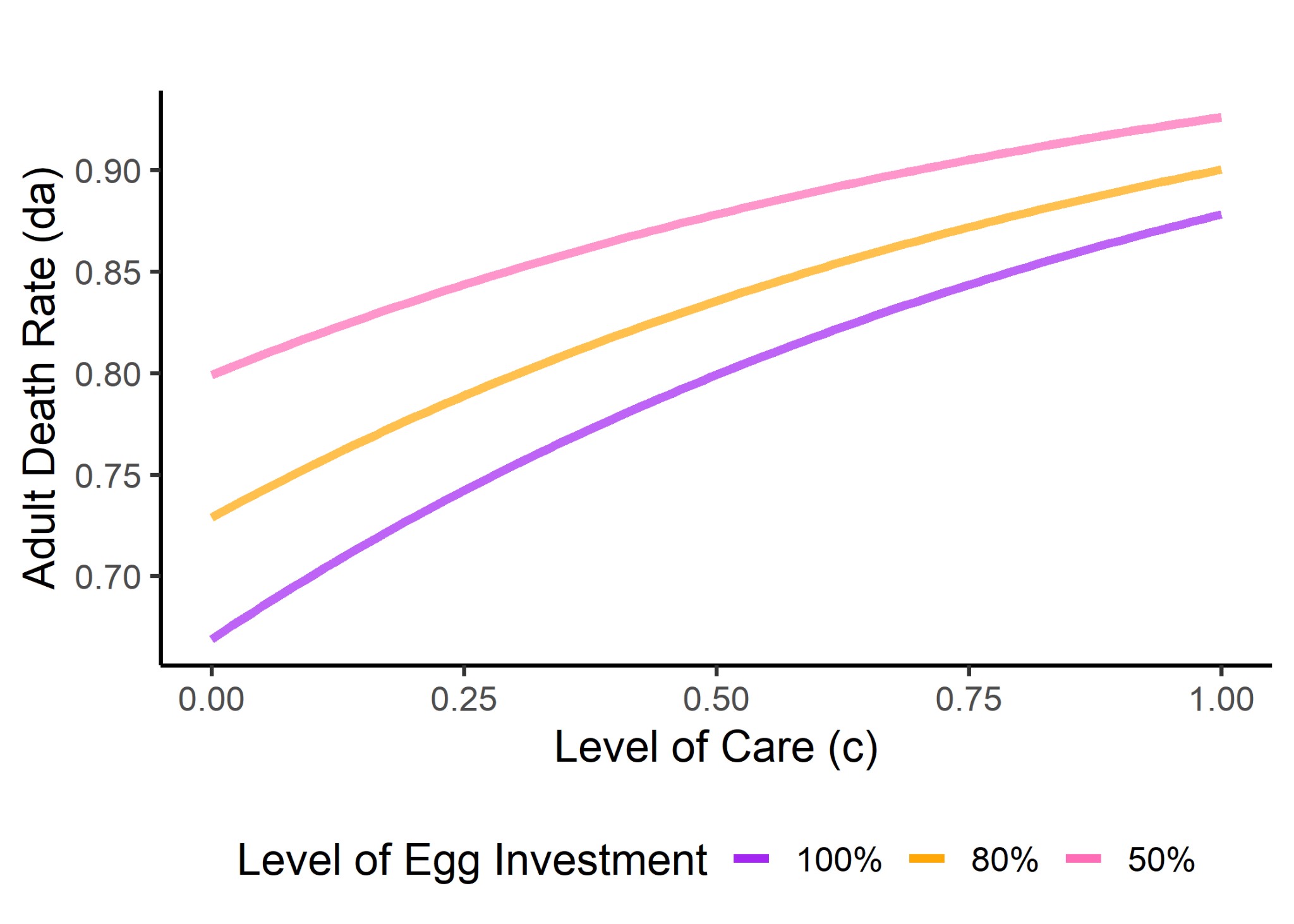


Figure S11. Benefit of additional care: additional care decreases egg death rate.


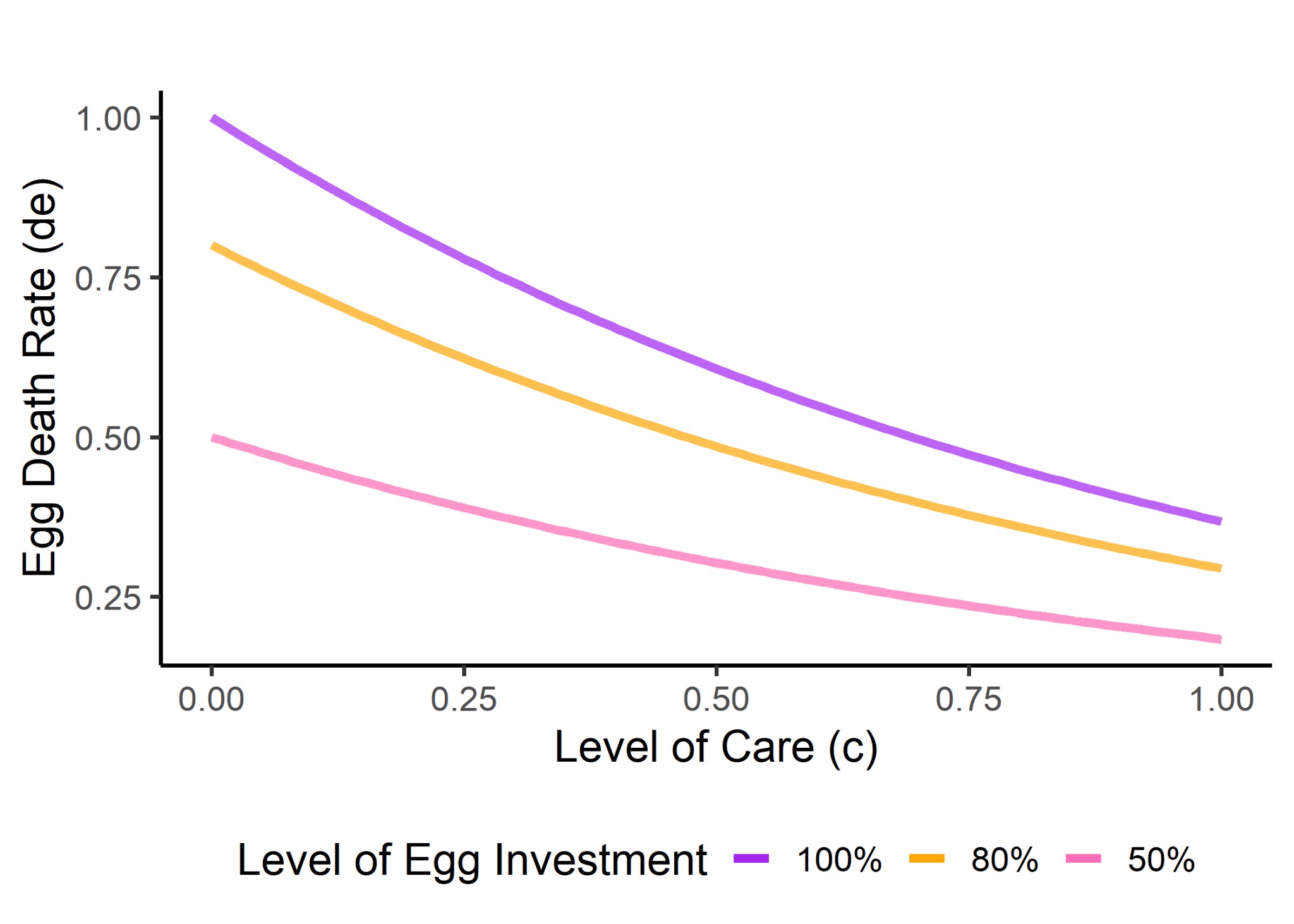


Figure S12. Benefit of additional care: additional care increases the fertilization rate


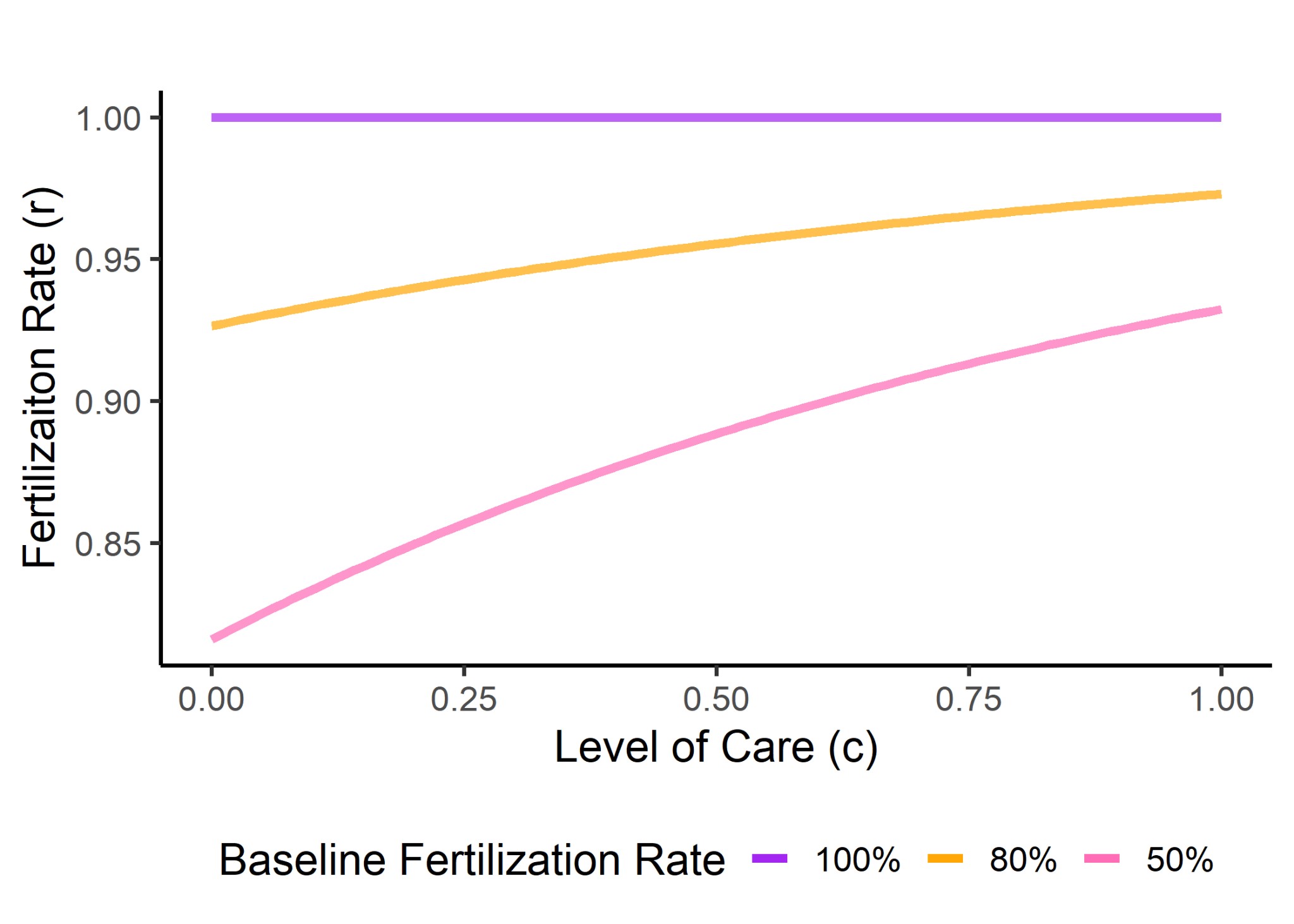


Figure S13. Cost of the mating trait: mating trait increases adult death rate.


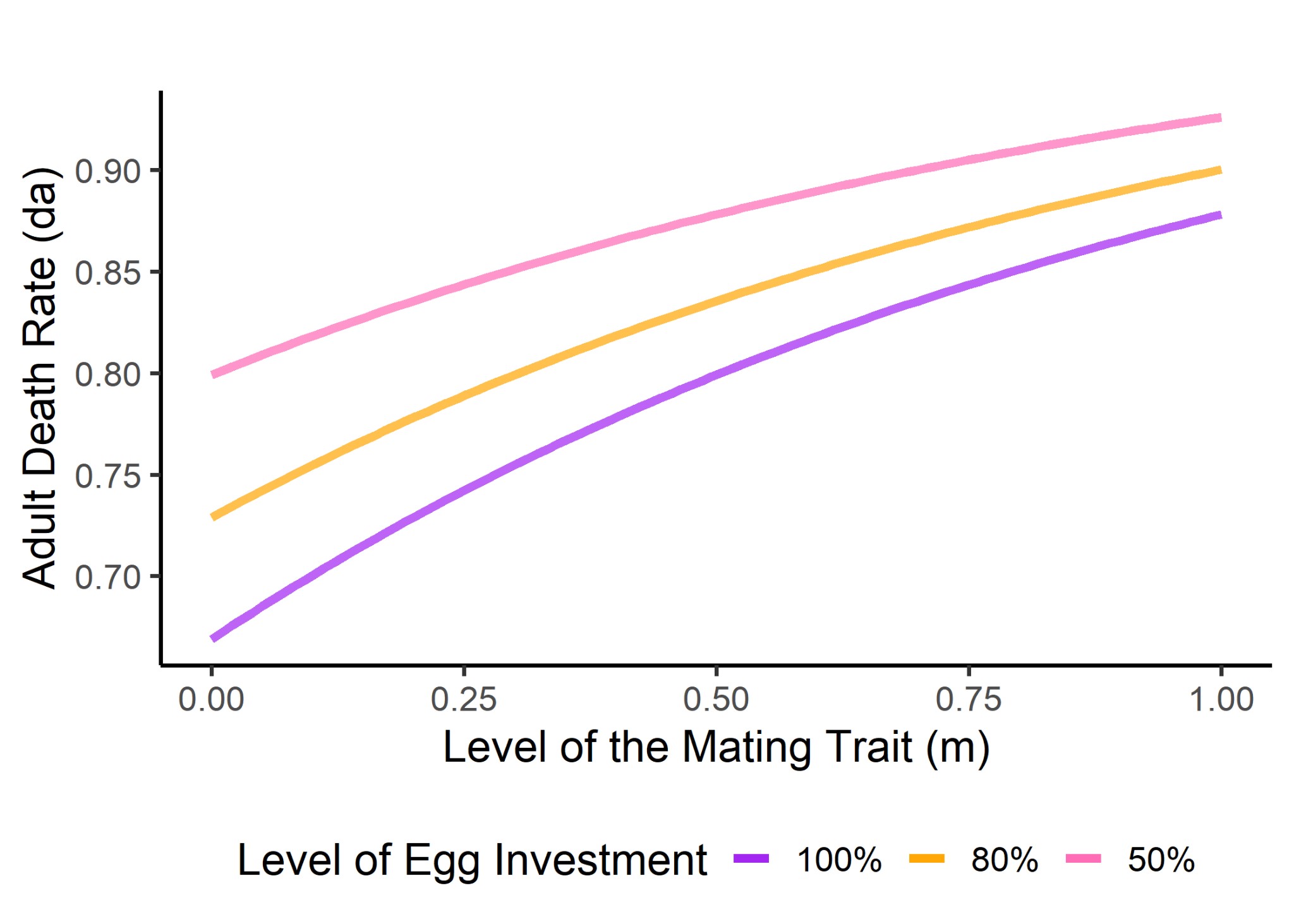


Figure S14. Benefit of the mating trait: mating trait increases fertilization rate.


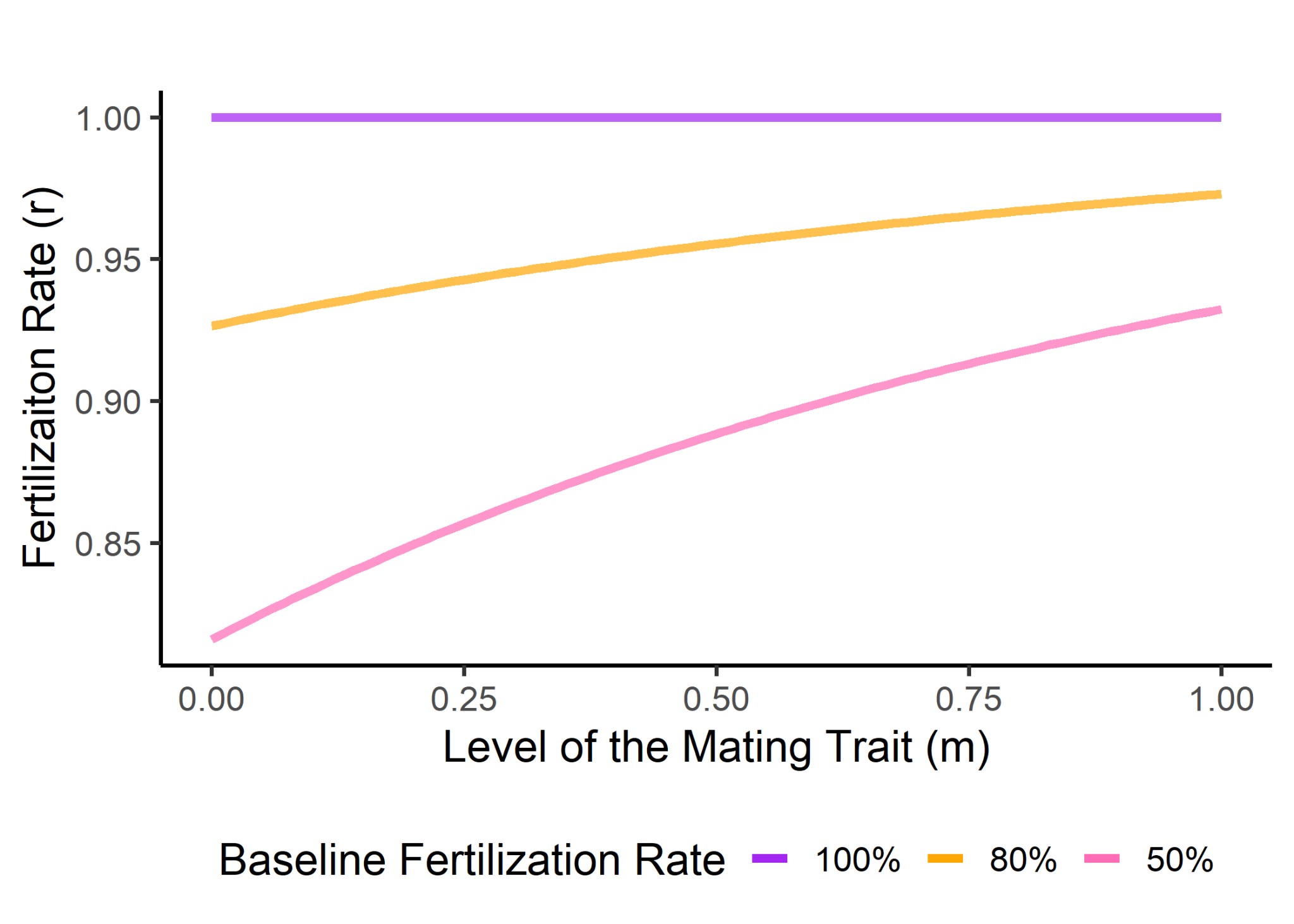

Supplement: Supplementary file 1 — Data S1. [file ECE3-14-e70189-s001.docx]
